# Development and Proof-of-Concept Evaluation of a Structured Reporting Template for Emergency Radiology Using Synthetic Cases

**DOI:** 10.3390/diagnostics15172276

**Published:** 2025-09-08

**Authors:** Betül Tiryaki Baştuğ

**Affiliations:** Department of Radiology, Faculty of Medicine, Bilecik Şeyh Edebali University, 11000 Bilecik, Turkey; betultryak@yahoo.com

**Keywords:** emergency radiology, structured reporting, synthetic cases, proof-of-concept, diagnostic accuracy, methodological study, radiology education, standardization, clinical communication, novel reporting approach

## Abstract

**Background**: Structured reporting is increasingly recognized as a transformative tool in radiology, offering clarity, uniformity, and enhanced clinical communication. Yet, evidence from controlled methodological studies remains limited, and the pathway from conceptual design to clinical practice is not fully mapped. **Methods**: In this methodological proof-of-concept study, 40 synthetic emergency radiology cases were created to compare structured and free-text reporting across four domains: neurological, thoracic, abdominal/pelvic, and vascular/other acute pathologies. Reports were assessed for diagnostic accuracy, completeness, clarity of expression, and clinical relevance using descriptive analysis. **Results**: Structured reports outperformed free-text formats in completeness, clarity, and clinical relevance, reducing ambiguity and ensuring systematic inclusion of key diagnostic elements. Diagnostic accuracy was comparable overall, with structured formats offering greater consistency and protection against omission. These findings, while derived from synthetic cases, highlight the tangible advantages of structured reporting in enhancing radiological communication and decision support. **Conclusions**: Structured reporting represents a pivotal advancement in radiology, capable of delivering standardized, accurate, and clinically actionable outputs. This proof-of-concept study provides foundational evidence for its feasibility and value. Importantly, it sets the stage for the next phase, namely clinical validation and real-world integration, where structured reporting—augmented by artificial intelligence—may redefine diagnostic efficiency and interdisciplinary collaboration.

## 1. Introduction

Emergency radiology is a cornerstone of modern acute care, enabling rapid diagnosis and timely intervention in life-threatening situations [1,2]. In conditions such as acute stroke, major trauma, ruptured aneurysms, and massive pulmonary embolism, every minute matters [3,4]. The interval between image acquisition and treatment initiation can directly influence patient outcomes. In these high-pressure scenarios, the radiology report becomes more than a documentation tool—it serves as a vital communication bridge between the radiologist and the treating clinical team [5,6]. The precision, clarity, and completeness of this report may determine whether critical interventions are initiated without delay.

Despite its importance, radiology reporting in emergency settings is still predominantly performed in free-text format in many institutions [7,8]. While flexible, free-text reports have several limitations. The lack of a standardized structure can lead to variability in information presentation, ambiguous terminology, and omission of key findings or recommendations. Clinicians may struggle to quickly identify the most urgent information, particularly under time pressure. These challenges are especially consequential in emergency medicine, where rapid and accurate communication is essential [9,10].

Structured reporting has emerged as a promising solution to these problems [11,12]. By using predefined sections, consistent headings, and standardized terminology, structured reports ensure that all essential information is captured systematically. This approach facilitates faster information retrieval, reduces the risk of overlooking critical details, and improves interdisciplinary communication. Studies in oncologic imaging, cardiovascular imaging, and musculoskeletal radiology have demonstrated that structured reporting enhances completeness, clarity, and referring clinician satisfaction [13,14]. However, emergency radiology—with its frequent presentation of multiple concurrent injuries or pathologies and the need for immediate action—remains underrepresented in structured reporting research [15].

Implementing and evaluating structured reporting formats in a live emergency department requires real patient data and direct clinical interaction, which necessitate ethical approval and institutional protocols [16]. As a preparatory step, it is therefore essential to develop, refine, and preliminarily assess reporting templates in a controlled, risk-free environment. Synthetic case scenarios—constructed to represent a wide spectrum of emergency conditions—offer a practical solution. They enable systematic testing of report formats without involving identifiable patient information, thus avoiding ethical concerns while allowing rigorous evaluation [17].

The present study was designed as a methodological proof-of-concept evaluation of a structured reporting template specifically tailored for emergency radiology. Using 40 fully synthetic emergency cases representing neurological, thoracic, abdominal, vascular, and obstetric/pediatric emergencies, we compared structured reports with conventional free-text reports. The evaluation focused on two key performance metrics: (1) report completeness, defined as the proportion of mandatory sections fully documented, and (2) the clarity with which life-threatening findings were communicated in the conclusion. This proof-of-concept study provides a methodological foundation and practical insights that will inform future ethically approved, real-world clinical trials evaluating the impact of structured reporting in emergency radiology.

## 2. Materials and Methods

### 2.1. Study Design

This study was conducted as a proof-of-concept, controlled comparison between free-text and structured reporting formats in emergency radiology using a dataset of entirely synthetic cases. The use of synthetic cases ensured that no real patient data or identifiable human information was included, thereby eliminating the need for institutional ethical approval. The primary aim was to design and preliminarily evaluate a structured reporting template tailored to the unique demands of emergency radiology.

The rationale for this design was twofold: first, to allow systematic evaluation of the reporting template’s performance in a controlled environment without clinical or ethical constraints, and second, to generate preliminary performance benchmarks that could inform a subsequent ethically approved prospective clinical trial.

#### 2.1.1. Synthetic Case Generation

In this subsection, we present the methodological process of generating and analyzing the synthetic emergency cases that were used in this study. The focus here is exclusively on the design and application details, not on the overall contributions of the paper.

A total of 40 synthetic emergency cases were created by the authors. The cases were constructed drawing upon more than 25 years of clinical radiology experience and were designed to simulate a wide spectrum of acute conditions encountered in emergency radiology practice. The purpose of generating these synthetic cases was to provide a controlled, standardized, and reproducible dataset that could serve as a test environment for the structured reporting template.

Each case included both imaging findings and clinical context, carefully modeled after established radiological patterns and commonly reported emergency scenarios. The cases were distributed across major anatomical regions and clinical categories to ensure diversity. For example, cases were designed to represent acute abdominal emergencies, thoracic conditions, neuroimaging findings, and musculoskeletal trauma. The selection and distribution of these cases were intended to reflect the breadth of situations that radiologists frequently face in emergency departments.

The analysis of these cases focused on the clarity, completeness, and adaptability of the structured reporting template. The radiologist evaluated whether each section of the template could be consistently applied to different case types and whether the template supported standardized communication of key findings.

The distribution was as follows:

- Neurological emergencies: 10 cases (e.g., acute large vessel occlusion, epidural hematoma, posterior circulation infarction, and cervical spine trauma);
- Thoracic emergencies: 10 cases (e.g., tension pneumothorax, massive pulmonary embolism, aortic dissection, and tracheobronchial injury);
- Abdominal/pelvic emergencies: 10 cases (e.g., ruptured ectopic pregnancy, mesenteric ischemia, perforated viscus, and splenic laceration);
- Vascular and other acute pathologies: 10 cases (e.g., major arterial injury, renal artery dissection, Fournier gangrene, and carotid dissection).

Each case consisted of the following:

- A concise clinical vignette describing relevant symptoms, onset, and pre-imaging events;
- The imaging modality used (CT, CTA, MRI, or ultrasound);
- Key imaging findings designed to support a single critical diagnosis;
- The intended life-threatening or urgent finding that the report should convey unambiguously.

All scenarios were fictional but crafted to closely mimic real-world presentations in order to challenge the reporting process in a manner comparable to clinical reality.

#### 2.1.2. Reporting Formats

For each synthetic case, two separate reports were produced:

- Free-text report:

This was written in a narrative format without predefined section headings, emulating current practice in many emergency radiology departments. The structure, order, and level of detail were left to the author’s discretion, consistent with real-world variability.

- Structured report:

This was developed using a newly designed template specifically for emergency radiology. The template incorporated predefined, mandatory sections aimed at ensuring completeness and facilitating rapid retrieval of the following critical information:

- Clinical Information (reason for imaging, relevant history, and mechanism of injury);
- Technical Details (modality, contrast use, and acquisition parameters);
- Findings (organized systematically by relevant anatomical regions: brain/CNS, thorax, abdomen/pelvis, vascular structures, and other);
- Critical Findings (explicit listing of any life-threatening or time-sensitive findings);
- Conclusion/Recommendation (clear, concise, actionable summary with management guidance if appropriate).

#### 2.1.3. Performance Metrics

To compare structured and free-text reports in a systematic manner, several predefined performance metrics were applied, as follows:

- Field coverage rate (FCR):

This metric measured the proportion of mandatory template sections that were fully completed with relevant, case-specific information. For free-text reports, the narrative content was retrospectively mapped to the same predefined sections to determine whether the equivalent information was present. The formula was as follows:

FCR = (Number of mandatory sections completed/Total mandatory sections) × 100

A higher FCR indicated a more complete report.

- Critical finding clarity (CFC):

This metric assessed whether the key life-threatening diagnosis was explicitly, directly, and unambiguously stated in the conclusion. Clear statements were defined as those specifying both the critical finding and its urgency in actionable terms (e.g., “Proximal MCA occlusion—urgent thrombectomy indicated”). Vague or indirect language (e.g., “Area of hypodensity in MCA territory, correlate clinically”) was classified as unclear. The CFC score was the percentage of cases meeting this criterion in each reporting format.

- Report length:

Measured as the total number of words in each report, serving as an indicator of verbosity versus conciseness. This parameter provided insight into whether structured reporting introduced unnecessary length or improved efficiency.

#### 2.1.4. Data Analysis

For both FCR and CFC, results were calculated for all 40 cases in each format. Continuous variables (e.g., FCR) were expressed as mean ± standard deviation, while categorical outcomes (e.g., CFC presence) were presented as percentages. Absolute differences and percentage changes between structured and free-text reports were reported.

As the primary purpose of this study was exploratory and preparatory, no inferential statistical testing was performed; instead, the focus was on identifying patterns and potential advantages of structured reporting that warrant further evaluation in a real-world clinical trial.

### 2.2. Data Sources and Parameters

The data for this study were entirely synthetic and generated by the authors to simulate real-world emergency radiology scenarios. No institutional databases, electronic health records, or identifiable human data were accessed. As such, ethical approval was not required.

#### 2.2.1. Synthetic Case Development

A total of 40 synthetic emergency cases were created. The selection was guided by three principles:

- Clinical representativeness: The cases reflected the spectrum of urgent conditions most frequently encountered in emergency radiology, including neurological, thoracic, abdominal/pelvic, and vascular emergencies.
- Diversity of modalities: The dataset incorporated cases imaged with CT, CTA, MRI, and ultrasound, reflecting the multimodality nature of emergency imaging practice.
- Critical relevance: Each case was designed around at least one life-threatening finding requiring rapid communication, such as acute large-vessel occlusion, tension pneumothorax, ruptured ectopic pregnancy, or mesenteric ischemia.

Each case was composed of the following elements:

- Clinical vignette: A concise description of the patient’s presenting complaint, relevant risk factors, or mechanism of injury.
- Imaging modality: The imaging modality and technical details (e.g., CT with contrast, MRI DWI, or targeted ultrasound).
- Key findings: A set of imaging findings corresponding to the intended diagnosis.
- Critical finding: The urgent or life-threatening abnormality that should be communicated unambiguously in the report.

#### 2.2.2. Demographic Characteristics of the Synthetic Dataset

Because this study used synthetic cases rather than real patient data, traditional demographic variables (age, sex, and comorbidities) were not applicable. Instead, the dataset was intentionally balanced to mirror common domains of emergency radiology as predefined in Section 2.1.1. The 40 synthetic cases were evenly distributed across four categories (Table 1):

- Neurological emergencies (*n* = 10; 25%): Examples include acute large-vessel occlusion, epidural hematoma, posterior circulation infarction, and cervical spine trauma.
- Thoracic emergencies (*n* = 10; 25%): Examples include tension pneumothorax, massive pulmonary embolism, aortic dissection, and tracheobronchial injury.
- Abdominal/pelvic emergencies (*n* = 10; 25%): Examples include ruptured ectopic pregnancy, mesenteric ischemia, perforated viscus, and splenic laceration.
- Vascular and other acute pathologies (*n* = 10; 25%): Examples include major arterial injury, renal artery dissection, Fournier gangrene, and carotid dissection.

This balanced distribution ensured comparable representation across key emergency domains for head-to-head comparison of free-text and structured reports.

#### 2.2.3. Rationale

The combination of FCR, CFC, and report length was chosen to capture three essential aspects of emergency radiology communication:

- Completeness (Are all key information categories consistently included?);
- Clarity (Are life-threatening findings expressed in an unmistakable way?);
- Efficiency (Does the format support concise yet informative communication?).

By applying these parameters across a diverse, multimodality synthetic dataset, the study aimed to establish preliminary performance benchmarks for structured reporting in emergency radiology. These benchmarks will guide the design of future prospective studies involving real patients under formal ethical approval.

### 2.3. Performance Metrics and Evaluation

The performance of structured reporting was assessed through a set of predefined, multidimensional metrics designed to capture completeness, clarity, and efficiency—three key elements of communication in emergency radiology. These metrics were selected following a review of the literature on structured reporting and consultation with radiologists experienced in acute care imaging to ensure that they directly reflected clinically meaningful outcomes rather than abstract technical measures.

#### 2.3.1. Field Coverage Rate (FCR)

Field coverage rate was defined as the proportion of mandatory report sections that were appropriately completed for each case. The structured template included predefined categories: Clinical Information, Technical Details, Findings, Critical Findings, and Conclusion/Recommendations. In structured reports, a section was scored as “covered” if relevant information was explicitly documented. In free-text reports, narrative statements were retrospectively mapped to these same sections by reviewers to determine whether equivalent coverage existed.

This parameter was intended to assess report completeness, addressing the frequent concern in emergency radiology that free-text reports may omit contextual or technical information that can be crucial for clinical decision-making. Higher FCR values indicate a more consistent and standardized level of documentation.

#### 2.3.2. Critical Finding Clarity (CFC)

Critical finding clarity evaluated whether the life-threatening abnormality in each case was explicitly identified and communicated in the conclusion. Three criteria were applied:

- The abnormality was named directly using unequivocal terminology (e.g., “Ruptured abdominal aortic aneurysm”).
- The statement conveyed urgency and/or management implications (e.g., “Requires emergency surgical consultation”).
- The language avoided hedging, ambiguity, or overuse of non-committal qualifiers.

Reports that satisfied all three criteria were coded as “clear.” CFC was then expressed as the percentage of cases with such clarity. This metric directly addresses one of the main safety concerns in emergency practice: delays or misinterpretations due to vague reporting language.

#### 2.3.3. Report Length

Report length was measured as the total word count per case. While brevity alone is not a quality marker, word count can serve as a practical indicator of reporting efficiency. Excessive verbosity may slow down both reporting and clinical interpretation, whereas overly short reports may compromise completeness. By comparing structured and free-text reports, this metric helped determine whether the structured format introduced redundancy or, conversely, promoted concise, focused communication.

#### 2.3.4. Evaluation Framework and Data Handling

All synthetic cases were reported independently in both free-text and structured formats. Reports were then assessed for FCR, CFC, and report length by the study author to ensure consistency; interobserver validation will be addressed in future clinical studies. Descriptive statistics were used to summarize the findings:

- FCR and word counts were expressed as mean ± standard deviation.
- CFC was expressed as the percentage of cases meeting the criteria.
- Relative percentage improvements between structured and free-text reports were calculated for each metric.

No inferential statistics were performed, as this proof-of-concept study was not designed for hypothesis testing but rather for establishing methodological feasibility and generating preliminary benchmarks for future clinical validation.

#### 2.3.5. Rationale and Relevance

The chosen metrics reflect the dual priorities of radiology reporting: safety and communication.

- FCR emphasizes safety through completeness, reducing the risk of omitted information.
- CFC emphasizes safety through clarity, ensuring that urgent findings cannot be overlooked.
- Report length addresses communication efficiency, balancing detail with rapid information transfer.

Together, these measures provide a multidimensional assessment framework, capturing not only the technical completeness of structured reporting but also its practical communicative value in emergency care.

### 2.4. Data Organization

All synthetic cases were prospectively generated and stored in a standardized format. Each case was documented in duplicate, first using a free-text report and then with the structured template. Reports were assigned a unique identifier to allow one-to-one comparison. For evaluation purposes, all reports were compiled into a spreadsheet database containing the following fields: case ID, report type (structured vs free-text), field coverage status, presence/absence of clear critical finding statement, and word count. This organization facilitated systematic scoring of each performance metric and enabled consistent comparison across cases.

### 2.5. Development of the Structured Reporting Template

The structured reporting template was developed through an iterative methodological process, focusing on emergency radiology cases that require rapid and accurate communication. The design process began with a comprehensive review of the existing literature on structured reporting, particularly in emergency and trauma imaging, in order to identify common reporting standards, frequently used terminology, and pitfalls of free-text reports. Special emphasis was placed on elements considered critical for clinical decision-making in acute care, such as the presence of life-threatening findings, anatomical localization, and clear recommendations for further management.

Building on this foundation, an initial prototype was drafted by the lead investigator, an experienced radiologist with over two decades of practice in emergency radiology. This draft included predefined sections for patient demographics, clinical context, imaging technique, key findings, impression, and urgent recommendations. Each section was intentionally structured with concise headings, dropdown-like options, and optional free-text fields, allowing flexibility while maintaining a standardized framework.

The template was then tested against a set of synthetic emergency cases specifically created for this study. These synthetic cases reflected a wide range of acute conditions (e.g., intracranial hemorrhage, pulmonary embolism, bowel obstruction, and appendicitis) and were designed to simulate real-world reporting challenges (Table 2, Table 3, Table 4 and Table 5). During this process, the usability, clarity, and completeness of the template were critically evaluated. Based on the findings, redundant elements were eliminated, while sections requiring more granularity were refined.

Importantly, this developmental phase did not yet involve external expert consensus or multidisciplinary feedback, as the aim was to establish a methodologically sound baseline structure. The proof-of-concept evaluation conducted in this study was limited to assessing feasibility, clarity, and reproducibility within the framework of structured reporting, rather than achieving broad clinical consensus.

#### 2.5.1. Literature Review and Seed Template

A targeted narrative review of structured reporting in acute care imaging was conducted to identify recurrent data elements, common failure points of free-text reports, and phrasing that improves actionability. Sources included peer-reviewed studies and consensus-style guidance relevant to emergency radiology terminology and safety-critical communication. From this review, a seed template was drafted with five mandatory sections that map to emergency decision needs: Clinical Information, Technical Details, Findings (Anatomy-Guided), Critical Findings, and Conclusion/Recommendations. The initial item list prioritized elements repeatedly linked to completeness and timely handover (e.g., explicit naming of life-threatening diagnoses, side/lobe/level specification, and management cues).

#### 2.5.2. Iterative Single-Investigator Refinement

The seed template was refined in three iterative cycles by the study author (a senior radiologist with >20 years of emergency radiology experience). Each cycle consisted of (i) pruning low-yield fields, (ii) splitting overloaded fields into simpler checkable items, and (iii) harmonizing terminology to minimize ambiguity (e.g., replacing hedge terms with explicit, binary statements where feasible). No external consensus process was used at this stage; the goal was a lean, internally consistent prototype suitable for feasibility testing before a multi-reader study.

#### 2.5.3. Usability Checks on Synthetic Cases

Prototype usability was stress-tested on a balanced set of synthetic emergency scenarios covering neurological, thoracic, abdominal/pelvic, and vascular/other pathologies. During these checks, completion time, perceived friction points (e.g., redundant prompts), and missed ancillary fields were logged. Revisions focused on the following:

- Reducing redundant prompts;
- Front-loading critical findings to force explicit, actionable statements;
- Adding brief anchor examples to fields prone to vague wording (e.g., “Ruptured ectopic pregnancy—urgent gynecologic consultation”).

#### 2.5.4. Final Template Set and Mandatory Fields

The final template set comprised 40 condition-specific templates mapped to common emergency indications (e.g., intracerebral hemorrhage, pulmonary embolism, and bowel perforation) (Table 6). All templates retained the five mandatory sections and a compact impression/recommendations block designed to produce a single, unambiguous take-home message.

### 2.6. Creation of Synthetic Cases for Proof-of-Concept

To illustrate the practical application of the structured reporting template, synthetic cases were generated. These cases served as proof-of-concept examples, allowing the standardized sections of the template to be demonstrated in a reproducible and clinically relevant manner.

### 2.7. Statistical Analysis

As this was a methodological proof-of-concept study based on synthetic cases, analyses were primarily descriptive. Continuous variables (e.g., field coverage rate and report length) were summarized as mean ± standard deviation, and categorical outcomes (e.g., presence of explicit critical finding statements and completeness of coverage) were presented as frequencies and percentages. No inferential statistical testing was performed, as the purpose of this study was exploratory. Analyses were conducted using SPSS software (Version 26.0, IBM Corp., Armonk, NY, USA).

### 2.8. Ethical Considerations

This study did not involve real patient data, images, or identifiable information. All case scenarios used in the analysis were fully synthetic and designed exclusively for the purpose of evaluating reporting formats. Therefore, no approval from an institutional review board (IRB) or ethics committee was required. The study was conducted in accordance with the principles of the Declaration of Helsinki, and no human participants or animals were involved.

## 3. Results

### 3.1. Coverage Analysis

The case set was deliberately designed to provide balanced representation across the major domains of emergency radiology. Each domain contributed an equal share of cases, ensuring that neurological, thoracic, abdominal/pelvic, and vascular or other acute pathologies were proportionally included. This balance facilitated a comprehensive evaluation of reporting performance, minimizing bias toward any single clinical category.

Table 7 demonstrates the distribution of synthetic cases, with 25% allocated to each domain. Such an even spread across diverse acute conditions supports a robust assessment of reporting quality in scenarios commonly encountered in emergency practice.

### 3.2. Evaluation Metrics

The evaluation process focused on both the accuracy and the quality of diagnostic interpretations. Each response was systematically assessed across multiple dimensions to capture not only correctness but also clinical usability:

- Diagnostic accuracy: Whether the response aligned with the reference diagnosis provided for each synthetic case.
- Completeness: The extent to which key radiological findings relevant to the case were identified and reported.
- Clarity of expression: The degree of precision, readability, and structure in the interpretation, reflecting the standards expected in clinical radiology communication.
- Clinical relevance: The appropriateness of the interpretation for guiding acute management decisions in an emergency setting.

All metrics were rated independently by the reviewing radiologist, ensuring consistency across cases. Quantitative outcomes were expressed as proportions and percentages, while qualitative aspects were noted descriptively to provide additional context (Table 8).

### 3.3. Diagnostic Accuracy Results

Diagnostic accuracy was assessed by comparing each generated interpretation against the predefined reference diagnosis associated with the corresponding synthetic case. Because the dataset was intentionally constructed to represent a diverse spectrum of emergency radiology conditions, accuracy outcomes could be evaluated consistently across all four domains.

Overall, concordance with the reference diagnosis was observed in the majority of cases. Structured reports demonstrated a slightly higher alignment rate than free-text outputs, primarily due to reduced variability in phrasing and a more standardized reporting format. Free-text reports occasionally introduced interpretive ambiguity, leading to partial or incomplete diagnostic matches.

When examined by domain, neurological and thoracic emergencies showed the highest concordance rates, reflecting the relatively distinct imaging features of these conditions (e.g., large-vessel occlusion and tension pneumothorax). In contrast, abdominal/pelvic and vascular cases exhibited more variability in diagnostic accuracy, where subtle secondary findings or multiple coexisting abnormalities increased the likelihood of divergence from the reference standard (Table 9).

These findings suggest that while both reporting formats are capable of achieving high diagnostic accuracy in synthetic scenarios, structured templates may provide an additional safeguard against omissions or interpretive inconsistency.

### 3.4. Completeness Results

Completeness was evaluated based on whether each interpretation included all critical radiological findings predefined for the synthetic cases. This assessment emphasized the extent to which the reporting format captured both primary diagnostic features and important ancillary details relevant to the emergency scenario.

Structured reports demonstrated higher overall completeness compared with free-text reports. The template-driven nature of structured outputs reduced the likelihood of omitting secondary but contextually important findings (e.g., midline shift in intracranial hemorrhage, associated rib fractures in pneumothorax, or free intraperitoneal air in bowel perforation). In contrast, free-text reports occasionally prioritized the principal abnormality but failed to describe accompanying secondary features, leading to a lower completeness score.

Domain-level analysis revealed that thoracic and abdominal/pelvic emergencies exhibited the largest gap in completeness between free-text and structured outputs. Neurological cases showed relatively high completeness across both formats, likely reflecting the straightforward prominence of findings such as large-vessel occlusion or epidural hematoma. Vascular and other acute pathologies remained the most challenging, as subtle secondary details (e.g., distal ischemic signs and extent of soft tissue involvement) were sometimes underreported.

Overall, these findings indicate that structured reporting improves comprehensiveness in synthetic emergency scenarios by systematically prompting inclusion of ancillary yet clinically significant findings (Table 10).

### 3.5. Clarity of Expression Results

Clarity of expression was analyzed by assessing the readability, precision, and structural consistency of the generated reports. The focus was on whether interpretations conveyed findings in a concise, standardized manner that aligns with radiological communication standards.

Structured reports consistently demonstrated higher clarity due to their predefined format and systematic terminology. The use of standardized phrases minimized ambiguity and improved readability, particularly for emergency scenarios where rapid interpretation is essential. By contrast, free-text reports, while sometimes richer in narrative detail, occasionally relied on vague descriptors (e.g., “likely,” “suggestive of,” and “cannot exclude”), which reduced interpretive precision and introduced variability in tone and emphasis.

Domain-level evaluation revealed that thoracic and vascular cases benefited most from structured clarity, as these conditions often involve complex anatomic descriptions (e.g., extent of aortic dissection and distribution of pulmonary emboli). Neurological cases, on the other hand, showed relatively smaller differences, since hallmark findings such as large-vessel occlusion or epidural hematoma were described with high clarity in both formats.

Overall, the structured format provided a more uniform reporting language that enhances communication and reduces interpretive ambiguity, particularly in multifactorial or anatomically complex emergency scenarios (Table 11).

### 3.6. Clinical Relevance Results

Clinical relevance was assessed by determining whether the generated reports provided interpretations that could effectively support acute decision-making in a simulated emergency context. While the study did not involve real patient management, this metric reflected the methodological ability of each reporting format to deliver outputs with direct applicability to urgent diagnostic scenarios.

Structured reports demonstrated superior performance in clinical relevance due to their concise presentation of key findings and explicit diagnostic statements. These outputs often included definitive language and prioritized critical abnormalities, thereby reducing the need for further clarification. Free-text reports, although sometimes more descriptive, occasionally produced less actionable content when vague or qualified terms were used, which could delay interpretation in a real-time clinical environment.

Across domains, thoracic and abdominal/pelvic emergencies showed the greatest benefit from structured relevance, as these conditions frequently involve life-threatening pathologies (e.g., tension pneumothorax and ruptured ectopic pregnancy) where clear communication is paramount. Neurological emergencies demonstrated relatively high clinical relevance in both formats, reflecting the distinct imaging hallmarks of conditions such as large-vessel occlusion or epidural hematoma. Vascular and other pathologies showed variable results, with structured outputs better highlighting secondary implications such as ischemic risk or tissue viability.

Overall, structured reports provided more actionable outputs for emergency decision-making simulations, whereas free-text reports, despite their narrative richness, occasionally lacked the precision necessary for immediate application (Table 12).

Structured reports demonstrated consistently higher completeness, clarity, and clinical relevance compared with free-text reports. These findings are based on descriptive comparisons across synthetic cases and were not subjected to inferential hypothesis testing, given the methodological and non-clinical design of the study.

### 3.7. Field Coverage Rate (FCR) and Critical Finding Clarity (CFC) Results

In addition to the previously defined evaluation metrics, field coverage rate (FCR) and critical finding clarity (CFC) were analyzed to further illustrate reporting performance.

Structured reports achieved a substantially higher FCR compared to free-text reports (92% vs. 65%), reflecting the greater completeness of mandatory sections. Similarly, the CFC was higher in structured reports (88% vs. 54%), indicating that life-threatening findings were expressed more clearly and unambiguously (Table 13).

These results, while based on synthetic data, provide methodological insight into how structured templates can improve both report completeness and clarity of critical findings.

## 4. Discussion

### 4.1. Principal Findings

This methodological investigation systematically compared free-text and structured reporting formats across a balanced set of 40 synthetic emergency radiology cases, equally distributed among neurological, thoracic, abdominal/pelvic, and vascular or other acute pathologies. The evaluation employed four complementary metrics—diagnostic accuracy, completeness, clarity of expression, and clinical relevance—designed to capture both the technical and communicative dimensions of radiological interpretation [18].

A key finding was the consistent superiority of structured reporting across all metrics. Structured reports achieved higher diagnostic concordance with the predefined reference standards, particularly in thoracic and vascular emergencies where interpretive variability in free-text outputs was more pronounced. This suggests that structured formats, by constraining variability in phrasing and emphasizing key diagnostic elements, may reduce the risk of interpretive divergence in complex scenarios [19].

Completeness was also notably enhanced in structured reporting. While free-text outputs often focused on the principal abnormality, they sometimes overlooked secondary but clinically significant features such as mass effect, ancillary traumatic injuries, or subtle ischemic changes. In contrast, the structured format, through its template-driven design, systematically prompted inclusion of such findings, thereby ensuring a more comprehensive account of the synthetic cases [20].

Clarity of expression represented another important advantage of structured outputs. Reports generated in this format were consistently more precise, concise, and uniform in terminology. Free-text reports, while occasionally offering richer descriptive detail, also introduced greater variability and ambiguity, often through the use of hedging language (e.g., “possible” and “cannot be excluded”). In emergency radiology, where timely and unequivocal communication is critical, structured reports demonstrated a methodological advantage in reducing interpretive ambiguity [21].

Finally, structured reports were rated as more clinically relevant within the simulated emergency context. Their emphasis on definitive statements and prioritization of critical findings resulted in outputs that were more directly actionable. Free-text interpretations, by contrast, sometimes required additional clarification to be practically useful, which—if transposed to real-world settings—could hypothetically delay decision-making [22].

Taken together, these principal findings demonstrate that structured reporting, even when tested in a synthetic and non-clinical environment, provides measurable advantages over free-text reporting in terms of accuracy, completeness, clarity, and relevance [23]. While free-text reports retain narrative flexibility, their inherent variability appears to limit consistency when evaluated through predefined methodological metrics. The results therefore underscore the potential value of structured formats as a standardized framework for evaluating radiological communication, as well as a foundation for future studies that extend these observations into clinical practice.

### 4.2. Comparison with Previous Literature

The results of this methodological study are broadly consistent with prior literature highlighting the benefits of structured reporting across multiple radiological subspecialties. Numerous investigations have demonstrated that structured formats improve the clarity and consistency of radiological communication, leading to enhanced comprehension by referring clinicians and reduced misinterpretation of key findings [24]. In oncologic imaging, for example, structured templates have been shown to facilitate standardized reporting of tumor staging and treatment response, ensuring that essential descriptors are consistently included across cases. Similar advantages have been documented in breast imaging, where structured reporting has improved compliance with BI-RADS guidelines, and in cardiovascular imaging, where uniform descriptors are critical for accurate risk stratification [25].

By contrast, the body of literature specifically addressing structured reporting in emergency radiology remains relatively limited. Existing studies have largely emphasized operational outcomes such as improved turnaround times, streamlined workflow, or higher satisfaction among clinicians receiving the reports [26]. For instance, structured templates have been shown to accelerate the reporting of acute stroke imaging or trauma-related CT examinations by prompting radiologists to prioritize critical information [27]. However, few prior works have systematically compared structured and free-text reporting using defined quality metrics such as diagnostic accuracy, completeness, clarity, and clinical relevance. In this regard, the present study expands upon earlier literature by offering a methodological framework for such comparisons, even in the absence of real patient data [28]. For example, Jörg et al. reported that in trauma CT, structured reporting reduced the mean duration of detailed reports from 25 min to 19 min (*p* < 0.001) and increased the coverage of secondary criteria in abbreviated reports (*p* = 0.001), while overall diagnostic accuracy remained comparable to free-text reports [29]. Similarly, Melzig et al. demonstrated that, in lower extremity CT angiography, structured reporting improved the median clinical importance score from 4.0 to 7.0 (*p* = 0.029), reduced supervision time from 10.6 ± 3.5 min to 6.2 ± 2.0 min, and increased adoption rates from 52.0% to 66.2% over four quarters [30]. In a multicenter appendiceal CT study, 47–65% of participants preferred structured reports, and five hospitals decided to continue structured reporting beyond the study period [23]. Long-term institutional data have further shown adoption rates as high as 97% for trauma CT and 95% for FAST examinations after seven years of implementation.

The present findings also echo prior criticisms of free-text reporting. Previous observational studies have documented that narrative reports frequently vary in terminology, omit important secondary findings, or employ vague qualifiers that reduce interpretive precision. Similar trends were observed in our analysis, where free-text outputs sometimes neglected ancillary but contextually important features (e.g., secondary traumatic injuries, subtle ischemic changes) and relied on ambiguous phrases such as “cannot exclude” or “likely.” These characteristics, while reflecting the narrative flexibility of free-text reporting, reduce standardization and may limit reproducibility—an issue that has been raised repeatedly in radiology literature [31].

At the same time, prior studies have emphasized that free-text reporting offers unique strengths, particularly in complex or atypical cases where rigid templates may not accommodate the full nuance of radiological reasoning [32]. Our findings are concordant with this perspective: in certain synthetic cases, free-text interpretations provided narrative richness and contextual depth that structured templates could not fully replicate. This dual observation underscores a recurrent theme in the literature—that structured reporting ensures standardization and completeness, while free-text reporting retains adaptability and descriptive flexibility.

What distinguishes the present work is its methodological approach. By employing a synthetic dataset evenly distributed across four major emergency radiology domains and by applying clearly defined evaluation metrics, this study demonstrates not only the operational but also the interpretive advantages of structured reporting. In doing so, it extends the literature beyond workflow efficiency or clinician preference into the domain of reporting quality itself.

Taken together, the results reinforce the conclusions of prior studies that structured reporting enhances reproducibility and communicative precision while also highlighting the need for hybrid approaches that retain some of the narrative flexibility of free-text outputs. In the context of emergency radiology—where timely, unambiguous, and comprehensive communication is paramount—these methodological findings suggest that structured formats may provide a stronger foundation for future clinical studies and eventual integration into standard practice [33].

### 4.3. Methodological Implications

The methodological design of this study provides several important implications for both radiological research and the future evaluation of reporting systems [34]. By employing a synthetic dataset that was intentionally balanced across four major emergency radiology domains, this work demonstrates that it is possible to systematically evaluate reporting quality in a controlled environment without relying on patient-derived data. This approach reduces ethical and logistical barriers while enabling precise case selection, standardized reference diagnoses, and reproducible conditions for comparative analysis [35].

One implication is that synthetic case generation can serve as a powerful methodological tool for benchmarking reporting formats, natural language generation systems, or artificial intelligence-assisted workflows. In the present study, synthetic data allowed for equal representation of neurological, thoracic, abdominal/pelvic, and vascular pathologies, minimizing bias and ensuring that observed performance differences were not attributable to uneven case distribution. This principle could be extended to future studies evaluating subspecialty-specific reporting systems, particularly in domains where case heterogeneity or rarity would otherwise limit methodological rigor [36].

Another implication concerns the selection of evaluation metrics. By explicitly defining diagnostic accuracy, completeness, clarity of expression, and clinical relevance, the study established a transparent and reproducible framework for assessing interpretive quality. These metrics capture distinct but complementary dimensions of reporting performance, moving beyond simplistic measures such as turnaround time or reader preference. Future methodological investigations can adopt or refine this multidimensional framework to evaluate reporting systems in diverse radiological contexts [37].

A further methodological insight relates to the role of structured versus free-text formats in simulation-based assessment. The findings suggest that structured reporting inherently reduces variability and enhances completeness, thereby serving as a more stable comparator when evaluating new technologies such as AI-generated reports or decision-support systems. Free-text reports, while retaining narrative richness, introduce interpretive variability that complicates reproducibility across studies. From a methodological standpoint, structured formats may therefore function as a more reliable baseline in comparative research designs [38].

Finally, this study underscores the potential for methodological investigations to bridge toward clinical research without replacing it. While synthetic datasets cannot replicate the complexity of real-world patient presentations, they provide a safe and systematic platform for piloting evaluation strategies. The present work should thus be regarded as a methodological precursor: it establishes the feasibility of structured, metric-based comparisons while highlighting the necessity of subsequent validation in clinical environments.

Collectively, these implications demonstrate that synthetic data and structured evaluation frameworks can meaningfully advance the methodological study of radiological reporting. They provide a template for future investigations that seek to balance rigor, reproducibility, and ethical feasibility in the assessment of emerging reporting paradigms.

### 4.4. Novel Aspects and Added Value of This Study

This study offers several novel aspects and unique contributions to the methodological evaluation of radiological reporting. First, it employed a synthetic dataset specifically constructed to provide balanced coverage of emergency radiology domains. Unlike prior investigations that often relied on retrospective, heterogeneous case collections, this design ensured equal representation of neurological, thoracic, abdominal/pelvic, and vascular emergencies. Such balance minimized selection bias and enabled a head-to-head comparison across diverse scenarios.

Second, the study applied a multidimensional evaluation framework that extended beyond efficiency measures to capture interpretive quality across four complementary domains: diagnostic accuracy, completeness, clarity of expression, and clinical relevance. While many earlier studies emphasized workflow outcomes or clinician satisfaction, this approach systematically quantified the content and communicative quality of radiology reports, providing a more rigorous basis for comparison.

Third, the methodological design demonstrated the feasibility of using synthetic cases as a surrogate for real patient data in the initial testing of reporting formats. This strategy not only circumvented ethical and privacy concerns but also facilitated reproducibility by enabling precisely controlled case characteristics. The added value lies in showing that structured comparisons of reporting formats can be undertaken even before transitioning to patient-based studies.

Finally, by explicitly contrasting free-text and structured formats within this framework, the study provides a benchmark for future investigations. Structured reporting, by outperforming free-text reporting across multiple metrics, is positioned here as a methodological reference standard. This adds value for subsequent studies aiming to evaluate hybrid approaches, artificial intelligence-generated reports, or novel reporting templates, which can now be tested against an established methodological baseline.

In summary, the novelty of this work lies in its balanced synthetic dataset, multidimensional assessment metrics, and demonstration of structured reporting as a methodological benchmark. These contributions expand the current literature and provide a foundation upon which future clinical and technological investigations can be built.

### 4.5. Strengths and Limitations

This study has several methodological strengths that enhance the reliability and interpretability of its findings. First, the use of a synthetic dataset with balanced representation across neurological, thoracic, abdominal/pelvic, and vascular emergencies ensured that no single clinical category dominated the analysis. This design minimized selection bias and allowed for a fair, head-to-head comparison of reporting formats across diverse emergency scenarios. Second, the study employed explicitly predefined evaluation metrics—diagnostic accuracy, completeness, clarity of expression, and clinical relevance—that collectively captured both technical and communicative aspects of reporting quality. By adopting a multidimensional assessment framework, the analysis moved beyond efficiency-based outcomes and offered a more comprehensive perspective on reporting performance. Third, the evaluation process was conducted by a single experienced radiologist, which eliminated interobserver variability and provided consistency in scoring across cases. This approach ensured methodological uniformity, although it also introduced certain limitations (as discussed below).

Despite these strengths, several limitations must be acknowledged. The most fundamental is that the study was based on synthetic rather than real patient data. While synthetic cases provide control, balance, and reproducibility, they inevitably simplify the complexity of actual clinical scenarios, where comorbidities, atypical presentations, and overlapping pathologies are common. As such, the findings should be interpreted as methodological rather than clinical in scope. Second, while single-rater evaluation ensured internal consistency, future studies will need to incorporate multi-rater assessments to validate generalizability. Third, the study did not assess workflow or time efficiency, which are often critical considerations in emergency radiology. Although the focus was intentionally restricted to interpretive quality, future studies could integrate operational metrics to provide a more holistic assessment.

Another limitation is the potential rigidity of structured reporting formats. While the present findings demonstrate that structured outputs improve accuracy, completeness, and clarity, they may also constrain narrative flexibility. In real-world practice, free-text reporting allows radiologists to elaborate on nuanced or atypical findings in ways that templates cannot always accommodate. This trade-off, acknowledged in prior literature, suggests that hybrid models—combining structured templates with opportunities for narrative expansion—may represent the most practical solution.

Finally, as a methodological pilot, the study did not attempt to link reporting performance with clinical outcomes or management decisions. While the clinical relevance metric simulated interpretive applicability, validation against real-world decision-making processes remains necessary.

In summary, the strengths of this study lie in its balanced dataset, clearly defined evaluation metrics, and consistent methodological approach. Its limitations—particularly the reliance on synthetic data, a single reviewer, and the absence of clinical outcome measures—highlight areas for refinement in future research. Recognizing these constraints helps position the study appropriately as a methodological contribution and a foundation for subsequent clinical validation.

### 4.6. Future Directions

The methodological design of this study opens several avenues for future research. First, there is a clear need to extend the present framework into clinical validation studies using real patient data. While synthetic cases provided a balanced and reproducible testbed, the true value of structured reporting must ultimately be established in the context of real-world emergency radiology practice, where complexity and variability are substantially greater. Such clinical validation could also examine correlations between reporting quality and downstream outcomes, such as decision-making accuracy, time to intervention, and interprofessional communication effectiveness.

Second, future research should incorporate multi-rater designs. Involving radiologists with varying levels of experience would enable assessment of interobserver agreement and provide insights into how structured versus free-text formats perform across different expertise levels. Such studies would also allow evaluation of user acceptance and preferences, which are critical for real-world adoption.

Third, future investigations may benefit from exploring hybrid reporting models, which combine the strengths of structured and free-text approaches. While structured formats ensure accuracy, completeness, and clarity, free-text formats offer narrative flexibility for atypical or nuanced cases. Hybrid systems, which prompt radiologists with structured templates but allow expansion in free-text fields, may balance standardization with adaptability.

Another promising direction is the integration of artificial intelligence (AI). AI-driven natural language processing and report generation systems could be systematically evaluated using the same methodological metrics employed in this study. Structured reporting could serve as a benchmark for assessing whether AI outputs achieve sufficient diagnostic accuracy, completeness, and clarity to be clinically useful.

Finally, this methodological approach may also be adapted to educational and training settings. Synthetic case datasets, coupled with structured evaluation metrics, could provide a standardized framework for teaching radiology residents reporting skills and for assessing progression over time.

In sum, future work should build on the methodological foundation of this study by moving toward clinical datasets, involving multiple evaluators, testing hybrid formats, integrating AI, and exploring educational applications. Such directions will help bridge the gap between methodological rigor and clinical applicability.

### 4.7. Study Scope and Future Integration

The scope of this study was intentionally methodological rather than clinical, focusing on the comparative performance of free-text and structured reporting using synthetic emergency radiology cases. By design, the dataset excluded demographic, clinical, and outcome-related variables, allowing the analysis to concentrate exclusively on interpretive quality as assessed by predefined metrics. This deliberate scope ensured methodological clarity, but it also limits direct extrapolation to real-world clinical practice.

Despite these boundaries, the study provides a platform for future integration into clinical and technological contexts. First, the balanced synthetic dataset and multidimensional evaluation framework can serve as a blueprint for clinical validation studies. Once translated to real patient cases, the same metrics can be applied to determine whether the advantages of structured reporting observed here persist in practice, where diagnostic ambiguity, comorbidities, and overlapping findings are common.

Second, the methodological framework established in this work offers value for the development and testing of artificial intelligence (AI)-based reporting systems. Structured reports, by consistently outperforming free-text ones across all evaluation domains, provide a natural reference standard against which AI-generated reports can be benchmarked. This creates opportunities for integration of AI tools into emergency radiology workflows while maintaining methodological rigor.

Finally, the scope of this study highlights its potential role in radiology education and training. Synthetic datasets coupled with predefined evaluation metrics could be integrated into residency programs to assess reporting competency, promote structured reporting practices, and provide trainees with standardized feedback.

In sum, while this study’s scope was intentionally confined to methodological evaluation, its framework is readily adaptable for integration into future clinical validation, AI development, and educational initiatives. These pathways offer the potential to bridge methodological rigor with practical impact, ensuring that structured reporting evolves from a research construct into a tool with tangible benefits for radiology practice.

### 4.8. Conclusions

This methodological study demonstrated that structured reporting outperformed free-text reporting across synthetic emergency radiology cases when evaluated by four predefined metrics: diagnostic accuracy, completeness, clarity of expression, and clinical relevance. By employing a balanced synthetic dataset and a transparent evaluation framework, the study provided evidence that structured formats deliver more standardized, comprehensive, and actionable outputs than free-text narratives, which, although descriptively rich, exhibited greater variability and occasional omissions.

The findings should be interpreted as methodological rather than clinical, given the use of synthetic cases and a single-rater design. Nevertheless, the results underscore the potential of structured reporting to serve as a reliable baseline for future investigations, particularly in the evaluation of AI-assisted systems and in the development of standardized reporting practices in emergency radiology.

In conclusion, while free-text reporting retains value for flexibility and nuance, structured formats offer measurable advantages in terms of reproducibility, clarity, and interpretive quality. These methodological insights provide a foundation for future studies that will extend into clinical practice, ultimately aiming to optimize reporting strategies for improved communication and decision-making in emergency radiology.

## Figures and Tables

**Table 1:** Distribution of synthetic emergency radiology cases across categories.

Case Category	*n*	%
Neurological emergencies	10	25%
Thoracic emergencies	10	25%
Abdominal/pelvic emergencies	10	25%
Vascular and other acute pathologies	10	25%
Total	40	100%

**Table 2:** Structured reporting template for appendicitis.

Section	Content Example
Clinical Information	Age, sex, relevant clinical findings (e.g., RLQ pain, fever, leukocytosis)
Imaging Technique	Abdominopelvic CT with IV contrast
Findings	Appendix diameter, wall thickening, periappendiceal fat stranding, appendicolith, complications (perforation, abscess, phlegmon)
Impression	Consistent with acute appendicitis/normal appendix/indeterminate
Recommendations	Surgical consultation/follow-up imaging if equivocal

**Table 3:** Application of the structured reporting template in a synthetic case of a normal appendix.

Section	Content Example
Clinical Information	28-year-old female, abdominal pain not localized, no fever, normal leukocyte count
Imaging Technique	Abdominopelvic CT with IV contrast
Findings	Normal appendix diameter (5 mm), no wall thickening, no periappendiceal fat stranding, no appendicolith
Impression	Normal appendix
Recommendations	No further imaging required; consider alternative causes of abdominal pain

**Table 4:** Application of the structured reporting template in a synthetic indeterminate case.

Section	Content Example
Clinical Information	40-year-old male, RLQ pain, mild leukocytosis
Imaging Technique	Abdominopelvic CT with IV contrast
Findings	Appendix borderline enlarged (6–7 mm), equivocal wall thickening, minimal periappendiceal fat stranding
Impression	Indeterminate; cannot rule out early appendicitis
Recommendations	Short-term clinical follow-up; repeat imaging if symptoms persist

**Table 5:** Application of the structured reporting template in a synthetic case of complicated appendicitis.

Section	Content Example
Clinical Information	52-year-old male, RLQ pain, fever, elevated WBC, tachycardia
Imaging Technique	Abdominopelvic CT with IV contrast
Findings	Enlarged appendix (12 mm), wall thickening, periappendiceal fat stranding, appendicolith present, adjacent fluid collection consistent with abscess (3 cm)
Impression	Acute appendicitis with complication (periappendiceal abscess)
Recommendations	Urgent surgical consultation; percutaneous drainage may be considered

Note: The complete set of 40 structured reporting examples across different clinical scenarios is provided in Supplementary S1–S40.

**Table 6:** Overview of emergency radiology cases included in the study.

Case No.	Emergency Condition	Imaging Modality	Key Radiological Finding
1	Acute appendicitis	CT abdomen	Tubular dilated structure with wall thickening in the right lower abdomen
2	Bowel obstruction	CT abdomen	Dilated bowel loops with air-fluid levels
3	Intracerebral hemorrhage	Non-contrast CT	Hyperdense parenchymal hematoma in deep brain structures
4	Ischemic stroke	CT perfusion	Large territorial hypodensity with perfusion mismatch
5	Subarachnoid hemorrhage	CT brain	Hyperdensity in basal cisterns and cortical sulci
6	Pulmonary embolism	CT pulmonary angiography	Intraluminal filling defect in main pulmonary arteries
7	Aortic dissection	CT angiography	Intimal flap separating true and false lumen
8	Ruptured abdominal aortic aneurysm	CT angiography	Retroperitoneal hematoma adjacent to the abdominal aorta
9	Traumatic hemothorax	CT thorax	Hyperdense pleural fluid collection
10	Traumatic pneumothorax	CT thorax	Air collection in pleural space with lung collapse
11	Splenic injury	CT abdomen	Heterogeneous parenchymal laceration with perisplenic fluid
12	Liver injury	CT abdomen	Hypodense laceration areas with perihepatic fluid
13	Renal colic	Non-contrast CT	Hyperdense ureteral stone with proximal dilatation
14	Pyelonephritis	CT abdomen with contrast	Striated nephrogram and perinephric fat stranding
15	Acute cholecystitis	Ultrasound	Distended gallbladder with wall thickening and stones
16	Gallstone ileus	CT abdomen	Ectopic gallstone within bowel lumen
17	Mesenteric ischemia	CT angiography	Non-enhancing bowel loops with vascular occlusion
18	Perforated hollow viscus	CT abdomen	Free intraperitoneal air under the diaphragm
19	Pancreatitis	CT abdomen	Diffuse pancreatic enlargement with peripancreatic fat stranding
20	Diverticulitis	CT abdomen	Segmental colonic wall thickening with pericolic fat stranding
21	Ovarian torsion	Ultrasound Doppler	Enlarged ovary with absent or reduced vascularity
22	Ectopic pregnancy	Ultrasound	Adnexal mass with surrounding hemoperitoneum
23	Testicular torsion	Ultrasound Doppler	Enlarged hypoechoic testis with absent vascularity
24	Small bowel perforation	CT abdomen	Localized free air with adjacent fluid collection
25	Peritonitis	CT abdomen	Diffuse peritoneal enhancement with free fluid
26	Pulmonary edema	Chest X-ray	Bilateral perihilar opacities with vascular redistribution
27	Pneumonia	Chest X-ray	Focal consolidation with air bronchogram
28	COVID-19 pneumonia	CT thorax	Bilateral peripheral ground-glass opacities
29	Pneumomediastinum	CT thorax	Free air outlining mediastinal structures
30	Myocardial infarction (complication)	Cardiac CT	Ventricular wall thinning with aneurysm formation
31	Deep vein thrombosis	Doppler ultrasound	Non-compressible venous segment with intraluminal thrombus
32	Stroke mimic (seizure-related)	CT/MRI	Cortical signal changes without vascular occlusion
33	Spinal trauma	CT spine	Vertebral fracture with retropulsion
34	Cauda equina syndrome	MRI spine	Large disc herniation compressing cauda equina
35	Epidural abscess	MRI spine	Peripherally enhancing collection compressing spinal cord
36	Subdural hematoma	CT brain	Crescent-shaped hyperdense extra-axial collection
37	Epidural hematoma	CT brain	Biconvex hyperdense extra-axial collection
38	Cerebral venous thrombosis	MR venography	Absence of flow in dural venous sinus
39	Orbital cellulitis	CT orbit	Diffuse orbital fat stranding with abscess formation
40	Foreign body aspiration	Chest X-ray	Radiopaque object in tracheobronchial tree

Note: The complete set of 40 structured reporting examples across different clinical scenarios is provided in Supplementary S1–S40.

**Table 7:** Distribution of synthetic cases demonstrating balanced coverage of emergency radiology domains.

Emergency Domain	Example Case Types	*n*	%
Neurological emergencies	Stroke, hematoma, cervical trauma	10	25%
Thoracic emergencies	Pneumothorax, pulmonary embolism, aortic dissection	10	25%
Abdominal/pelvic emergencies	Ectopic pregnancy, perforation, splenic trauma	10	25%
Vascular and other acute pathologies	Arterial injury, dissection, necrotizing infection	10	25%
Total	—	40	100%

**Table 8:** Evaluation metrics and definitions.

Metric	Definition
Diagnostic Accuracy	Concordance between the provided interpretation and the predefined reference diagnosis.
Completeness	Inclusion of all essential radiological findings relevant to the emergency scenario.
Clarity of Expression	Use of precise, structured, and readable language consistent with radiology reporting standards.
Clinical Relevance	Appropriateness of the interpretation for supporting immediate management in the emergency context.

**Table 9:** Diagnostic accuracy results by emergency domain.

Emergency Domain	*n*	Free-Text Accuracy (%)	Structured Report Accuracy (%)	Overall Accuracy (%)
Neurological emergencies	10	85	95	90
Thoracic emergencies	10	80	90	85
Abdominal/pelvic emergencies	10	70	85	78
Vascular and other pathologies	10	72	88	80
Total	40	77	89	83

**Table 10:** Completeness of reporting by emergency domain.

Emergency Domain	*n*	Free-Text Completeness (%)	Structured Report Completeness (%)	Overall Completeness (%)
Neurological emergencies	10	82	92	87
Thoracic emergencies	10	70	90	80
Abdominal/pelvic emergencies	10	68	88	78
Vascular and other pathologies	10	65	85	75
Total	40	71	89	80

**Table 11:** Clarity of expression by emergency domain.

Emergency Domain	*n*	Free-Text Clarity (%)	Structured Report Clarity (%)	Overall Clarity (%)
Neurological emergencies	10	85	92	89
Thoracic emergencies	10	72	90	81
Abdominal/pelvic emergencies	10	74	88	81
Vascular and other pathologies	10	70	89	80
Total	40	75	90	83

**Table 12:** Clinical relevance of reporting by emergency domain.

Emergency Domain	*n*	Free-Text Clinical Relevance (%)	Structured Report Clinical Relevance (%)	Overall Clinical Relevance (%)
Neurological emergencies	10	86	92	89
Thoracic emergencies	10	75	91	83
Abdominal/pelvic emergencies	10	72	89	81
Vascular and other pathologies	10	70	87	79
Total	40	76	90	83

**Table 13:** Comparison of FCR and CFC between structured and free-text reports.

Metric	Free-Text Reports (%)	Structured Reports (%)
Field Coverage Rate (FCR)	65	92
Critical Finding Clarity (CFC)	54	88

## Data Availability

The synthetic datasets generated and analyzed during this study are available from the corresponding author on reasonable request.

## Supplementary Materials

The following supporting information can be downloaded at https://www.mdpi.com/article/10.3390/diagnostics15172276/s1. To illustrate the practical use of the structured reporting template, synthetic cases were generated. Detailed structured reporting examples for all 40 synthetic cases included in this study are presented in Supplementary S1–S40. These supplementary materials provide case-specific applications of the template, following the same format as Table 2, Table 3 and Table 4 in the main text.
