# Supplementary material for: Development and Proof-of-Concept Evaluation of a Structured Reporting Template for Emergency Radiology Using Synthetic Cases
Source: Diagnostics (Basel). 2025 Sep 8;15(17):2276. doi: 10.3390/diagnostics15172276 (PMC12428108; doi:10.3390/diagnostics15172276)
Supplement: Supplementary file 1 [file diagnostics-15-02276-s001.zip › diagnostics-3855674-supplementary.pdf]

The complete set of 40 structured reporting examples across different clinical scenarios

S1- Structured Reporting Template for Appendicitis

Table 1. Structured Reporting Template for Appendicitis

Section	Content Example
Clinical Information	Age, sex, relevant clinical findings (e.g., RLQ pain, fever, leukocytosis)
Imaging Technique	Abdominopelvic CT with IV contrast
Findings	Appendix diameter, wall thickening, periappendiceal fat stranding, appendicolith, complications (perforation, abscess, phlegmon)
Impression	Consistent with acute appendicitis / Normal appendix / Indeterminate
Recommendations	Surgical consultation / Follow-up imaging if equivocal

Table 2. Application of the structured reporting template in a synthetic case of a normal appendix

Section	Content Example
Clinical Information	28-year-old female, abdominal pain not localized, no fever, normal leukocyte count
Imaging Technique	Abdominopelvic CT with IV contrast
Findings	Normal appendix diameter (5 mm), no wall thickening, no periappendiceal fat stranding, no appendicolith
Impression	Normal appendix
Recommendations	No further imaging required; consider alternative causes of abdominal pain

Table 3. Application of the structured reporting template in a synthetic indeterminate case

Section	Content Example
Clinical Information	40-year-old male, RLQ pain, mild leukocytosis
Imaging Technique	Abdominopelvic CT with IV contrast
Findings	Appendix borderline enlarged (6–7 mm), equivocal wall thickening, minimal periappendiceal fat stranding
Impression	Indeterminate; cannot rule out early appendicitis
Recommendations	Short-term clinical follow-up; repeat imaging if symptoms persist

Table 4. Application of the structured reporting template in a synthetic case of complicated appendicitis

Section	Content Example
Clinical Information	52-year-old male, RLQ pain, fever, elevated WBC, tachycardia
Imaging Technique	Abdominopelvic CT with IV contrast
Findings	Enlarged appendix (12 mm), wall thickening, periappendiceal fat stranding, appendicolith present, adjacent fluid collection consistent with abscess (3 cm)
Impression	Acute appendicitis with complication (periappendiceal abscess)
Recommendations	Urgent surgical consultation; percutaneous drainage may be considered

S2-Structured Reporting for Bowel Obstruction

Table 1. Structured Reporting Template for Bowel Obstruction

Section	Content Example
Clinical Information	Age, sex, symptoms (abdominal pain, distension, vomiting, constipation), relevant history (prior surgery, hernia, malignancy)
Imaging Technique	Abdominopelvic CT with IV/oral contrast (or non-contrast CT if contraindicated); abdominal X-ray if available
Findings	Bowel dilatation (diameter, location: small vs. large bowel), air-fluid levels, transition point (site and cause), bowel wall thickening, presence of pneumatosis or portal venous gas, peritoneal free fluid or free air
Impression	Small bowel obstruction / Large bowel obstruction / Indeterminate
Recommendations	Surgical consultation if high-grade or complicated; conservative management if partial/low-grade; follow-up imaging if equivocal

Table 2. Application of the structured reporting template in a synthetic case of small bowel obstruction

Section	Content Example
Clinical Information	65-year-old male, history of prior abdominal surgery, presenting with abdominal distension, vomiting, and constipation
Imaging Technique	Abdominopelvic CT with IV contrast
Findings	Dilated proximal small bowel loops (up to 4 cm) with multiple air-fluid levels, transition point identified at mid-ileum due to adhesive band, no free intraperitoneal air, no signs of ischemia
Impression	Small bowel obstruction, likely adhesive etiology, without complication
Recommendations	Surgical consultation; conservative management may be attempted if clinically stable

Table 3. Application of the structured reporting template in a synthetic indeterminate case

Section	Content Example
Clinical Information	54-year-old female, abdominal pain and bloating, no prior surgical history
Imaging Technique	Abdominopelvic CT with IV contrast
Findings	Mild dilatation of small bowel loops (up to 3 cm), equivocal transition zone in distal ileum, no wall thickening, no free air or ascites
Impression	Indeterminate; cannot confidently exclude evolving partial obstruction
Recommendations	Short-term clinical and imaging follow-up; consider repeat CT or contrast study if symptoms worsen

Table 4. Application of the structured reporting template in a synthetic case of complicated bowel obstruction

Section	Content Example
Clinical Information	72-year-old male, acute abdominal pain, distension, leukocytosis, elevated lactate
Imaging Technique	Abdominopelvic CT with IV contrast
Findings	High-grade small bowel obstruction with transition at terminal ileum caused by obstructing mass; bowel wall thickening, decreased mural enhancement, pneumatosis intestinalis, and small amount of portal venous gas
Impression	Complicated small bowel obstruction due to obstructing mass, with features concerning for ischemia
Recommendations	Urgent surgical consultation; management for suspected ischemic bowel

S3-Structured Reporting for Intracerebral Hemorrhage

Table 1. Structured Reporting Template for Intracerebral Hemorrhage

Section	Content Example
Clinical Information	Age, sex, presenting symptoms (sudden headache, focal neurological deficit, altered consciousness, hypertension history, anticoagulant use)
Imaging Technique	Non-contrast brain CT (first-line); CT angiography or MRI if needed
Findings	Hemorrhage location (lobar, deep, cerebellar, brainstem), size (dimensions or volume), shape, mass effect (midline shift, herniation), intraventricular extension, associated edema, underlying lesion (vascular malformation, tumor)
Impression	Intracerebral hemorrhage / No hemorrhage / Indeterminate
Recommendations	Neurology/neurosurgery consultation; repeat imaging for monitoring; further vascular imaging if secondary cause suspected

Table 2. Application of the structured reporting template in a synthetic case of lobar ICH

Section	Content Example
Clinical Information	68-year-old male, history of hypertension, sudden right-sided weakness
Imaging Technique	Non-contrast brain CT
Findings	Left parietal intraparenchymal hemorrhage measuring 3.2 × 2.8 cm, mild surrounding edema, no intraventricular extension, minimal mass effect without midline shift
Impression	Left parietal lobar intracerebral hemorrhage, likely hypertensive
Recommendations	Neurology and neurosurgery consultation; blood pressure control; follow-up CT within 24 hours

Table 3. Application of the structured reporting template in a synthetic indeterminate case

Section	Content Example
Clinical Information	55-year-old female, sudden headache, on oral anticoagulation
Imaging Technique	Non-contrast brain CT
Findings	Small hyperdense focus (5 mm) in right frontal lobe, equivocal between calcification vs. small acute hemorrhage, no mass effect
Impression	Indeterminate; possible small right frontal hemorrhage vs. calcification
Recommendations	Correlation with MRI susceptibility-weighted imaging; short-term follow-up CT if symptoms persist or worsen

Table 4. Application of the structured reporting template in a synthetic case of complicated ICH

Section	Content Example
Clinical Information	74-year-old male, acute coma, history of hypertension and atrial fibrillation
Imaging Technique	Non-contrast brain CT
Findings	Large left basal ganglia hemorrhage (5.5 × 4.8 cm) with intraventricular extension, significant surrounding edema, 8 mm midline shift, early uncal herniation
Impression	Complicated intracerebral hemorrhage with intraventricular extension and mass effect
Recommendations	Emergent neurosurgical consultation; consider external ventricular drain; intensive care monitoring

S4-Structured Reporting for Ischemic Stroke

Table 1. Structured Reporting Template for Ischemic Stroke

Section	Content Example
Clinical Information	Age, sex, clinical onset (sudden weakness, speech disturbance, visual changes), risk factors (hypertension, diabetes, atrial fibrillation), time from symptom onset
Imaging Technique	Non-contrast CT (NCCT); CT angiography (CTA) of head and neck; CT perfusion (CTP) or MRI (DWI, MRA, perfusion) if available
Findings	NCCT: early ischemic signs (loss of gray–white matter differentiation, sulcal effacement); ASPECTS score; CTA: site of arterial occlusion (ICA, MCA, ACA, PCA, basilar), collateral status; CTP/MRI: infarct core and penumbra volumes; hemorrhagic transformation if present
Impression	Acute ischemic stroke / No evidence of ischemia / Indeterminate
Recommendations	Urgent neurology consultation; IV thrombolysis/thrombectomy eligibility assessment; follow-up imaging if indeterminate

Table 2. Application of the structured reporting template in a synthetic case of normal study

Section	Content Example
Clinical Information	59-year-old female, acute left-sided weakness, onset 2 hours ago, atrial fibrillation
Imaging Technique	NCCT brain; CTA head and neck
Findings	NCCT: no acute hemorrhage, preserved gray–white differentiation, ASPECTS 10; CTA: no large vessel occlusion
Impression	No evidence of acute ischemia or hemorrhage
Recommendations	Clinical correlation; repeat imaging if symptoms persist; consider MRI if available

Table 3. Application of the structured reporting template in a synthetic indeterminate case

Section	Content Example
Clinical Information	65-year-old male, acute right arm weakness, onset 5 hours ago, history of hypertension
Imaging Technique	NCCT brain; CTA head and neck
Findings	NCCT: subtle loss of gray–white matter differentiation in left insula; ASPECTS 9; CTA: no proximal large vessel occlusion
Impression	Indeterminate for early ischemia; possible subtle left MCA territory changes
Recommendations	MRI with DWI for confirmation; close neurological monitoring; repeat CT in 6–12 hours

Table 4. Application of the structured reporting template in a synthetic case of large-vessel occlusion

Section	Content Example
Clinical Information	72-year-old male, acute global aphasia and right hemiplegia, onset 90 minutes ago
Imaging Technique	NCCT brain; CTA head and neck; CT perfusion
Findings	NCCT: early ischemic changes in left MCA territory, ASPECTS 7; CTA: left M1 MCA occlusion; CTP: infarct core 15 mL, penumbra 80 mL
Impression	Acute left MCA ischemic stroke due to large-vessel occlusion
Recommendations	Immediate thrombectomy evaluation; IV thrombolysis if within therapeutic window; urgent neurology and interventional radiology consultation

S5-Structured Reporting for Subarachnoid Hemorrhage (SAH)

Table 1. Structured Reporting Template for Subarachnoid Hemorrhage

Section	Content Example
Clinical Information	Age, sex, presenting symptoms (sudden severe headache, vomiting, loss of consciousness, seizure), clinical suspicion of aneurysm/trauma
Imaging Technique	Non-contrast CT brain ± CT angiography (if aneurysm suspected)
Findings	Subarachnoid blood distribution (basal cisterns, sylvian fissures, interhemispheric fissure, cortical sulci); Intraventricular extension; Hydrocephalus; Associated hematomas (intraparenchymal, subdural); Vascular abnormalities (aneurysm, AVM if CTA performed)
Impression	No hemorrhage / Mild SAH / Extensive SAH ± complications
Recommendations	Urgent neurosurgical/neuroradiology consultation; CTA/MRA for aneurysm detection if not yet performed; ICU monitoring

Table 2. Application of the structured reporting template in a synthetic case of normal CT

Section	Content Example
Clinical Information	47-year-old female, acute severe headache, suspected SAH
Imaging Technique	Non-contrast CT brain
Findings	No subarachnoid blood; basal cisterns and sulci clear; no hydrocephalus
Impression	Normal CT brain, no evidence of SAH
Recommendations	If suspicion remains high, lumbar puncture or CTA may be considered

Table 3. Application of the structured reporting template in a synthetic indeterminate case

Section	Content Example
Clinical Information	59-year-old male, sudden-onset headache, transient confusion
Imaging Technique	Non-contrast CT brain
Findings	Subtle hyperdensity in the left sylvian fissure, equivocal for small volume SAH; no hydrocephalus; no intraparenchymal bleed
Impression	Indeterminate; possible small focal SAH
Recommendations	CTA to exclude aneurysm; close clinical follow-up; repeat CT if symptoms worsen

Table 4. Application of the structured reporting template in a synthetic case of complicated SAH

Section	Content Example
Clinical Information	62-year-old female, sudden collapse, severe headache, GCS 7
Imaging Technique	Non-contrast CT brain and CT angiography
Findings	Extensive SAH in basal cisterns and sylvian fissures; intraventricular extension with hydrocephalus; associated left MCA aneurysm (8 mm) on CTA
Impression	Extensive SAH with intraventricular extension and hydrocephalus; ruptured left MCA aneurysm
Recommendations	Urgent neurosurgical/neuroradiology intervention (aneurysm clipping/coiling); external ventricular drain for hydrocephalus; ICU care

S6-Structured Reporting for Pulmonary Embolism

Table 1. Structured Reporting Template for Pulmonary Embolism

Section	Content Example
Clinical Information	Age, sex, risk factors (immobilization, surgery, malignancy, DVT), clinical findings (dyspnea, chest pain, hypoxia, tachycardia)
Imaging Technique	CT pulmonary angiography (CTPA) with IV contrast
Findings	Location of embolus (main, lobar, segmental, subsegmental pulmonary arteries), clot burden, right ventricular (RV) dilatation, interventricular septal bowing, pulmonary infarction, pleural effusion, alternative diagnoses
Impression	Pulmonary embolism present / absent / indeterminate
Recommendations	Urgent clinical correlation; anticoagulation if confirmed; echocardiography for RV strain if indicated; further imaging if equivocal

Table 2. Application of the structured reporting template in a synthetic case of central PE

Section	Content Example
Clinical Information	62-year-old female, acute dyspnea, history of prolonged immobilization
Imaging Technique	CT pulmonary angiography with IV contrast
Findings	Filling defect in the right main pulmonary artery extending into lobar branches; mild RV dilatation (RV/LV ratio >1); no pulmonary infarction
Impression	Acute central pulmonary embolism with evidence of RV strain
Recommendations	Urgent anticoagulation; cardiology and pulmonology consultation; echocardiographic correlation

Table 3. Application of the structured reporting template in a synthetic indeterminate case

Section	Content Example
Clinical Information	47-year-old male, chest pain and tachycardia, low clinical probability
Imaging Technique	CT pulmonary angiography with IV contrast
Findings	Motion artifact in lower lobes; equivocal filling defect in left lower lobe segmental artery; no RV dilatation; lungs otherwise clear
Impression	Indeterminate for pulmonary embolism (artifact vs. small PE)
Recommendations	Consider repeat CTPA with optimized technique; D-dimer correlation; clinical risk stratification

Table 4. Application of the structured reporting template in a synthetic case of complicated PE

Section	Content Example
Clinical Information	70-year-old male, sudden collapse, hypotension, history of atrial fibrillation
Imaging Technique	CT pulmonary angiography with IV contrast
Findings	Saddle embolus straddling the pulmonary trunk bifurcation with extension into both main pulmonary arteries; marked RV dilatation with septal bowing; bilateral small pleural effusions
Impression	Massive pulmonary embolism with hemodynamic compromise
Recommendations	Immediate resuscitation; urgent thrombolysis or thrombectomy; ICU admission

S7-Structured Reporting for Aortic Dissection

Table 1. Structured Reporting Template for Aortic Dissection

Section	Content Example
Clinical Information	Age, sex, acute chest/back pain, syncope, hypertension, connective tissue disorder (e.g., Marfan), hemodynamic status
Imaging Technique	CT angiography (CTA) of thorax and abdomen with arterial phase; ECG-gated CTA if available
Findings	Location of intimal flap (ascending, arch, descending aorta); Stanford classification (A or B); involvement of branch vessels (coronaries, carotids, mesenteric, renal, iliac); true vs. false lumen; presence of thrombosis, aneurysm, rupture, pericardial/pleural effusion
Impression	Stanford type A/B aortic dissection / No dissection / Indeterminate
Recommendations	Immediate cardiovascular surgery consultation (if type A); medical management vs. endovascular repair (if type B); follow-up CTA if indeterminate

Table 2. Application of the structured reporting template in a synthetic case of normal study

Section	Content Example
Clinical Information	60-year-old male, acute chest pain radiating to back, hypertension
Imaging Technique	CTA thorax and abdomen with arterial phase
Findings	Normal thoracic and abdominal aorta; no intimal flap, aneurysm, or periaortic hematoma
Impression	No evidence of aortic dissection
Recommendations	Consider alternative causes of chest pain; clinical correlation

Table 3. Application of the structured reporting template in a synthetic indeterminate case

Section	Content Example
Clinical Information	55-year-old female, tearing chest pain, history of hypertension
Imaging Technique	CTA thorax and abdomen
Findings	Subtle linear filling defect in proximal descending thoracic aorta; differentiation between motion artifact and intimal flap uncertain; no periaortic hematoma
Impression	Indeterminate; possible limited intimal flap vs. artifact
Recommendations	ECG-gated CTA or repeat imaging; close hemodynamic monitoring

Table 4. Application of the structured reporting template in a synthetic case of confirmed dissection

Section	Content Example
Clinical Information	68-year-old male, sudden chest pain radiating to back, hypotension, Marfan syndrome
Imaging Technique	CTA thorax and abdomen with arterial phase
Findings	Intimal flap in ascending aorta extending to arch and descending thoracic aorta; Stanford type A dissection; true and false lumen clearly visualized; pericardial effusion present; involvement of left renal artery
Impression	Acute Stanford type A aortic dissection with pericardial effusion
Recommendations	Immediate surgical consultation; high-risk of rupture; intensive care admission

S8-Structured Reporting for Ruptured Abdominal Aortic Aneurysm (rAAA)

Table 1. Structured Reporting Template for Ruptured Abdominal Aortic Aneurysm

Section	Content Example
Clinical Information	Age, sex, symptoms (acute abdominal/back pain, hypotension, syncope), risk factors (hypertension, smoking, atherosclerosis, known AAA)
Imaging Technique	Contrast-enhanced CT Angiography of abdomen and pelvis
Findings	Aneurysm size and location (infrarenal, juxtarenal, suprarenal); Presence of rupture signs (retroperitoneal hematoma, active contrast extravasation, discontinuity of aneurysm wall); Hemoperitoneum; Iliac artery involvement; Status of visceral arteries (renal, SMA, IMA); Other incidental findings
Impression	No aneurysm / Intact aneurysm / Ruptured AAA with/without active extravasation
Recommendations	Urgent vascular surgery consultation; Endovascular repair (EVAR) or open surgery depending on anatomy; Hemodynamic stabilization and ICU transfer

Table 2. Application of the structured reporting template in a synthetic normal case

Section	Content Example
Clinical Information	60-year-old male, abdominal pain, history of hypertension, no hypotension
Imaging Technique	Contrast-enhanced CT Angiography
Findings	Abdominal aorta normal caliber (2.0 cm); no aneurysm, no hematoma, no extravasation
Impression	Normal abdominal aorta; no evidence of aneurysm
Recommendations	No intervention required; manage alternative causes of abdominal pain

Table 3. Application of the structured reporting template in a synthetic indeterminate case

Section	Content Example
Clinical Information	68-year-old male, back pain, borderline hypotension, smoker
Imaging Technique	Contrast-enhanced CT Angiography
Findings	Abdominal aortic aneurysm measuring 5.4 cm infrarenal; mural thrombus present; no definite extravasation but subtle retroperitoneal stranding noted
Impression	Indeterminate for rupture; aneurysm at risk with suspicious periaortic changes
Recommendations	Close monitoring; urgent vascular surgery consult; repeat imaging if hemodynamic status worsens

Table 4. Application of the structured reporting template in a synthetic case of ruptured AAA

Section	Content Example
Clinical Information	74-year-old male, severe abdominal/back pain, hypotension, history of hypertension and smoking
Imaging Technique	Contrast-enhanced CT Angiography
Findings	Large infrarenal abdominal aortic aneurysm measuring 8.2 cm; active contrast extravasation into retroperitoneum; large retroperitoneal hematoma extending into left psoas; bilateral common iliac arteries patent
Impression	Ruptured abdominal aortic aneurysm with active bleeding and retroperitoneal hematoma
Recommendations	Immediate vascular surgery consultation; urgent EVAR if anatomically feasible, otherwise open surgical repair; aggressive hemodynamic stabilization in ICU

S9-Structured Reporting for Traumatic Hemothorax

Table 1. Structured Reporting Template for Traumatic Hemothorax

Section	Content Example
**Clinical Information**	Age, sex, mechanism of trauma (blunt/penetrating), hemodynamic status, respiratory distress, analgesia/anticoagulant use.
**Imaging Technique**	CT thorax with IV contrast (arterial/portal venous if vascular injury suspected); chest X-ray if CT unavailable.
**Findings**	Pleural fluid location (right/left/bilateral), distribution (dependent, loculated), maximal pleural stripe thickness (cm), estimated volume; attenuation/Hounsfield Units (acute blood typically >30–35 HU); fluid–fluid level; signs of **active extravasation** (contrast blush within pleural space); associated injuries: rib/sternal/scapular fractures, lung contusion/laceration, pneumothorax (hemopneumothorax), diaphragmatic injury, mediastinal hematoma; mass effect (atelectasis, mediastinal shift).
**Impression**	Traumatic hemothorax (side, extent: small/moderate/large) ± pneumothorax; **No hemothorax**; **Indeterminate** (e.g., poor opacification/beam hardening).
**Recommendations**	Trauma surgery/thoracic surgery consultation; tube thoracostomy if moderate/large or respiratory compromise; CTA if active bleeding suspected; follow-up imaging post-drain placement; analgesia and pulmonary hygiene.

Table 2. Application of the structured reporting template in a synthetic "no hemothorax" case

Section	Content Example
**Clinical Information**	35-year-old male, blunt chest trauma (MVC), pleuritic pain, stable vitals.
**Imaging Technique**	CT thorax with IV contrast.
**Findings**	No pleural fluid; lungs clear aside from minimal dependent atelectasis; no pneumothorax; no rib or sternal fractures; mediastinum unremarkable.
**Impression**	**No hemothorax or pneumothorax.** No acute osseous injury identified.
**Recommendations**	Clinical observation and analgesia as needed; no immediate imaging follow-up required unless symptoms progress.

Table 3. Application of the structured reporting template in a synthetic indeterminate case

Section	Content Example
**Clinical Information**	58-year-old female, fall from height, mild dyspnea, on antiplatelet therapy.
**Imaging Technique**	CT thorax with IV contrast.
**Findings**	Small dependent left pleural collection with attenuation **~25–30 HU** (borderline for acute blood); motion/beam-hardening artifact near diaphragm limits HU accuracy; no pneumothorax; nondisplaced fractures of left 7–8th ribs; no mediastinal hematoma; no clear contrast extravasation.
**Impression**	**Indeterminate small left pleural effusion vs. low-attenuation hemothorax.** Limited by motion/artifact.
**Recommendations**	Short-interval clinical reassessment; consider repeat CT or bedside ultrasound to characterize pleural fluid; manage pain and encourage pulmonary hygiene; low threshold for tube thoracostomy if respiratory status worsens.

Table 4. Application of the structured reporting template in a synthetic complicated hemothorax

Section	Content Example
**Clinical Information**	69-year-old male, penetrating chest trauma, hypotension, tachypnea.
**Imaging Technique**	CTA thorax (arterial phase).

Section	Content Example
**Findings**	**Large right pleural collection** (maximal thickness 6.5 cm) with heterogeneous high attenuation **45–70 HU** and **focal contrast blush** along the right posterolateral chest wall consistent with **active intercostal arterial extravasation**; compressive atelectasis of right lower lobe with mild mediastinal shift to left; right 5–7th rib fractures; small apical pneumothorax (hemopneumothorax).
**Impression**	**Massive right traumatic hemothorax with active arterial bleeding** and small concomitant pneumothorax; multiple rib fractures.
**Recommendations**	**Emergent tube thoracostomy** and hemodynamic resuscitation; urgent **interventional radiology embolization** of suspected intercostal source vs. operative control per trauma protocol; ICU admission; post-drain radiograph/CT to assess residual collection and lung re-expansion.

S10- Structured Reporting Template for Traumatic Pneumothorax

Table 1. Structured Reporting Template for Traumatic Pneumothorax

Section	Content Example
**Clinical Information**	Age, sex, mechanism of trauma (e.g., blunt chest trauma, penetrating injury), clinical symptoms (dyspnea, chest pain, hypoxia, hypotension)
**Imaging Technique**	Chest CT (with/without IV contrast) / Chest X-ray
**Findings**	Presence and size of pneumothorax (small/moderate/large), laterality (right/left/bilateral), presence of tension features (mediastinal shift, diaphragmatic depression), rib fractures, associated hemothorax, pulmonary contusion, subcutaneous emphysema
**Impression**	Traumatic pneumothorax (specify side, size, and if tension pneumothorax is present)
**Recommendations**	Urgent chest tube insertion if large/tension pneumothorax; close monitoring for small pneumothorax; surgical consultation if associated injuries present

Table 2. Application of the structured reporting template in a synthetic case of a small traumatic pneumothorax

Section	Content Example
**Clinical Information**	25-year-old male, blunt chest trauma from motor vehicle accident, mild dyspnea
**Imaging Technique**	Chest CT without IV contrast
**Findings**	Small right-sided apical pneumothorax (<10%), no mediastinal shift, right 6th rib fracture, minimal subcutaneous emphysema
**Impression**	Small right-sided traumatic pneumothorax, no tension features
**Recommendations**	Observation with supplemental oxygen; repeat imaging in 6–12 hours

Table 3. Application of the structured reporting template in a synthetic case of a large traumatic pneumothorax

Section	Content Example
**Clinical Information**	34-year-old male, penetrating stab wound to the left chest, severe dyspnea, tachycardia
**Imaging Technique**	Chest CT with IV contrast
**Findings**	Large left-sided pneumothorax (~50%), mediastinal shift to the right, diaphragmatic depression, moderate hemothorax, multiple rib fractures
**Impression**	Large left-sided traumatic pneumothorax with tension features
**Recommendations**	Urgent chest tube insertion; surgical consultation

Table 4. Application of the structured reporting template in a synthetic case of bilateral traumatic pneumothorax

Section	Content Example
**Clinical Information**	45-year-old male, fall from height, hypoxia, chest pain
**Imaging Technique**	Chest CT with IV contrast
**Findings**	Bilateral pneumothorax (right ~20%, left ~15%), associated pulmonary contusions, bilateral rib fractures, extensive subcutaneous emphysema
**Impression**	Bilateral traumatic pneumothoraces with associated pulmonary contusions
**Recommendations**	Bilateral chest tube insertion; intensive care monitoring

S11- Structured Reporting for Splenic Injury

Table 1. Structured Reporting Template for Splenic Injury

Section	Content Example
**Clinical Information**	Age, sex, mechanism of trauma (blunt/penetrating), hemodynamic status, left upper quadrant pain, associated injuries (left lower rib fractures), antithrombotic use.
**Imaging Technique**	Contrast-enhanced **CT abdomen/pelvis** (portal venous ± arterial phase if vascular injury suspected).
**Findings**	Spleen size and contour; **Laceration depth/length** (cm), **subcapsular/intraparenchymal hematoma** (% surface area or cm), **parenchymal devascularization**, **vascular injury** (pseudoaneurysm, **active contrast extravasation**), **hilar injury**; **Hemoperitoneum** extent/distribution (perisplenic, perihepatic, paracolic gutters, pelvis); associated injuries (left kidney, pancreas, ribs, diaphragm); incidental findings.
**Impression**	**Splenic injury present/absent/indeterminate**; **Estimated AAST grade (2018)** if applicable (I–V); presence/absence of vascular injury or active bleeding.
**Recommendations**	Trauma/surgery consult; **non-operative management (NOM)** for stable low–intermediate grade; **angiography/embolization** for vascular injury or active extravasation; **urgent surgery** if unstable or hilar/devastating injury; follow-up CT per protocol.

Table 2. Application of the structured reporting template in a synthetic "no injury" case

Section	Content Example
**Clinical Information**	33-year-old male, blunt abdominal trauma (MVC), stable vitals.
**Imaging Technique**	Contrast-enhanced CT abdomen/pelvis (portal venous phase).
**Findings**	Spleen normal in size and homogeneous enhancement; **no laceration or hematoma**; no perisplenic fluid; no left rib fractures; remaining solid organs unremarkable.
**Impression**	**No splenic injury** identified.
**Recommendations**	Clinical observation; no imaging follow-up required unless symptoms evolve.

Table 3. Application of the structured reporting template in a synthetic indeterminate case

Section	Content Example
**Clinical Information**	58-year-old female, fall from height, left flank pain; on antiplatelet therapy.
**Imaging Technique**	Contrast-enhanced CT abdomen/pelvis.
**Findings**	Linear **low-attenuation** focus (≤1 cm) at superior pole—**contusion vs. beam-hardening artifact**; thin **subcapsular fluid** crescent (≤5 mm) without mass effect; **no active extravasation**; trace pelvic fluid.
**Impression**	**Indeterminate** for minor splenic injury (**possible AAST grade I** contusion); no CT signs of active bleeding.
**Recommendations**	Short-interval clinical reassessment; consider **repeat CT** or ultrasound if pain worsens or hemoglobin drops; NOM if stable.

Table 4. Application of the structured reporting template in a synthetic complicated splenic injury

Section	Content Example
**Clinical Information**	47-year-old male, blunt trauma (handlebar), tachycardia, falling hemoglobin.
**Imaging Technique**	Dual-phase CT abdomen/pelvis (arterial + portal venous).
**Findings**	**Deep parenchymal lacerations** involving lower pole with **parenchymal devascularization** (~30%); **contrast blush** on arterial phase with pooling on portal venous phase = **active extravasation**; moderate **hemoperitoneum** (perisplenic, left paracolic gutter, pelvis); left 9–10th rib fractures; pancreas/kidney intact.

Section	Content Example
**Impression**	**High-grade splenic injury with active bleeding — estimated AAST grade IV** (laceration with vascular injury).
**Recommendations**	**Urgent IR angiography and splenic artery embolization**; NOM if hemodynamically stabilized post-embolization; surgical backup per trauma protocol; follow-up CT to assess devascularization/collection.

S12- Structured Reporting Template for Liver Injury

Table 1. Structured Reporting Template for Liver Injury

Section	Content Example
**Clinical Information**	Age, sex, trauma mechanism (e.g., blunt abdominal trauma, penetrating trauma), hemodynamic status
**Imaging Technique**	Contrast-enhanced abdominopelvic CT (arterial and portal venous phases)
**Findings**	Liver laceration or contusion, hematoma, vascular injury (active extravasation, pseudoaneurysm), biliary injury, hemoperitoneum, AAST grade
**Impression**	No liver injury / Indeterminate / Liver injury with grade (AAST I–VI)
**Recommendations**	Conservative management / Interventional radiology (embolization) / Surgical consultation

Table 2. Application of the structured reporting template in a synthetic case of a normal liver

Section	Content Example
**Clinical Information**	25-year-old male, blunt abdominal trauma after motor vehicle accident, hemodynamically stable
**Imaging Technique**	Contrast-enhanced abdominopelvic CT (arterial and portal venous phases)
**Findings**	Normal liver parenchyma, no laceration, no hematoma, no vascular abnormality, no free fluid
**Impression**	No liver injury
**Recommendations**	No further imaging required; clinical observation as indicated

Table 3. Application of the structured reporting template in a synthetic indeterminate case

Section	Content Example
**Clinical Information**	34-year-old female, fall from height, mild abdominal pain, stable vitals
**Imaging Technique**	Contrast-enhanced abdominopelvic CT (arterial and portal venous phases)
**Findings**	Subtle hypodense linear area in right hepatic lobe, questionable laceration vs artifact, no hemoperitoneum
**Impression**	Indeterminate; possible minor liver laceration (AAST I)
**Recommendations**	Short-term clinical and laboratory follow-up; consider repeat imaging if symptoms worsen

Table 4. Application of the structured reporting template in a synthetic case of complicated liver injury

Section	Content Example
**Clinical Information**	45-year-old male, blunt abdominal trauma from motorbike accident, hypotension, tachycardia
**Imaging Technique**	Contrast-enhanced abdominopelvic CT (arterial and portal venous phases)
**Findings**	Large laceration in right hepatic lobe (>10 cm, involving >3 segments), active contrast extravasation (arterial bleed), large perihepatic hematoma, moderate hemoperitoneum
**Impression**	Severe liver injury (AAST IV) with active bleeding
**Recommendations**	Urgent surgical consultation; interventional radiology embolization if feasible

S13- Structured Reporting Template for Renal Colic

Table 1. Structured Reporting Template for Renal Colic

Section	Content Example
**Clinical Information**	Age, sex, presenting symptoms (e.g., flank pain, hematuria, nausea/vomiting), prior stone history
**Imaging Technique**	Non-contrast CT of kidneys, ureters, bladder (CT KUB)
**Findings**	Presence/absence of urinary tract calculi, location (renal, ureteral, bladder), size (mm), degree of obstruction (hydronephrosis, hydroureter), perinephric stranding, secondary signs (delayed nephrogram, urinoma)
**Impression**	No evidence of urinary tract stone / Indeterminate / Stone detected (with location and obstruction grade)
**Recommendations**	Symptomatic treatment and follow-up / Urology consultation / Consider intervention (e.g., ureteroscopy, stent, lithotripsy)

Table 2. Application of the structured reporting template in a synthetic normal case

Section	Content Example
**Clinical Information**	30-year-old male, left flank pain, microscopic hematuria
**Imaging Technique**	Non-contrast CT KUB
**Findings**	No urinary tract calculi, normal renal parenchyma, no hydronephrosis, no perinephric stranding
**Impression**	No evidence of urinary tract stone
**Recommendations**	No specific imaging follow-up required; consider non-urologic causes of flank pain

Table 3. Application of the structured reporting template in a synthetic indeterminate case

Section	Content Example
**Clinical Information**	42-year-old female, right flank pain, nausea, trace hematuria
**Imaging Technique**	Non-contrast CT KUB
**Findings**	Punctate 2 mm hyperdensity in distal right ureter, equivocal for calculus vs vascular calcification; mild fullness of right collecting system
**Impression**	Indeterminate; possible tiny distal ureteral calculus
**Recommendations**	Short-term follow-up with repeat CT or ultrasound if symptoms persist; urology referral if clinical suspicion remains high

Table 4. Application of the structured reporting template in a synthetic complicated case

Section	Content Example
**Clinical Information**	55-year-old male, severe left flank pain, fever, leukocytosis
**Imaging Technique**	Non-contrast CT KUB
**Findings**	9 mm obstructing calculus at left proximal ureter, marked hydronephrosis, perinephric fat stranding, delayed nephrogram, perinephric fluid collection suggesting urinoma
**Impression**	Obstructing left proximal ureteral calculus with secondary infection (complicated renal colic)
**Recommendations**	Urgent urology consultation; consider percutaneous nephrostomy or ureteral stent placement; intravenous antibiotics

S14- Structured Reporting Template for Pyelonephritis

Table 1. Structured Reporting Template for Pyelonephritis

Section	Content Example
**Clinical Information**	Age, sex, presenting symptoms (e.g., fever, flank pain, dysuria), laboratory results (leukocytosis, pyuria, positive urine culture)
**Imaging Technique**	Contrast-enhanced CT abdomen and pelvis (corticomedullary and nephrographic phases)
**Findings**	Renal size, cortical hypodensities (wedge-shaped, striated nephrogram), perinephric fat stranding, renal/perinephric abscess, emphysematous changes, hydronephrosis, obstruction, vascular complications (renal vein thrombosis)
**Impression**	No evidence of pyelonephritis / Indeterminate / Pyelonephritis (uncomplicated or complicated)
**Recommendations**	Antibiotic therapy / Urology or interventional radiology consultation / Follow-up imaging in complicated cases

Table 2. Application of the structured reporting template in a synthetic normal case

Section	Content Example
**Clinical Information**	29-year-old female, fever, dysuria, positive urine culture (E. coli)
**Imaging Technique**	Contrast-enhanced CT abdomen and pelvis
**Findings**	Normal renal size and parenchymal enhancement, no focal hypodensity, no perinephric stranding
**Impression**	No imaging evidence of pyelonephritis
**Recommendations**	Continue clinical and laboratory follow-up; antibiotic treatment based on culture results

Table 3. Application of the structured reporting template in a synthetic indeterminate case

Section	Content Example
**Clinical Information**	37-year-old male, fever, left flank pain, leukocytosis
**Imaging Technique**	Contrast-enhanced CT abdomen and pelvis
**Findings**	Subtle ill-defined hypodense area in the mid-portion of the left kidney; minimal perinephric fat stranding; findings could represent early pyelonephritis versus artifact
**Impression**	Indeterminate; possible early pyelonephritis
**Recommendations**	Clinical correlation and antibiotic therapy; consider repeat CT or MRI if symptoms persist or worsen

Table 4. Application of the structured reporting template in a synthetic complicated case

Section	Content Example
**Clinical Information**	62-year-old diabetic female, high fever, left flank pain, septic parameters elevated
**Imaging Technique**	Contrast-enhanced CT abdomen and pelvis
**Findings**	Enlarged left kidney with multiple wedge-shaped cortical hypodensities, perinephric fat stranding, intraparenchymal abscess (2.5 cm), small pockets of gas consistent with emphysematous pyelonephritis
**Impression**	Complicated left pyelonephritis with abscess formation and emphysematous changes
**Recommendations**	Urgent urology consultation; intravenous antibiotics; consider percutaneous drainage or nephrectomy if deterioration

S15- Structured Reporting Template for Acute Cholecystitis

Table 1. Structured Reporting Template for Acute Cholecystitis

Section	Content Example
**Clinical Information**	Age, sex, symptoms (RUQ pain, fever, nausea/vomiting), lab values (leukocytosis, elevated CRP, abnormal LFTs)
**Imaging Technique**	Abdominal ultrasound / CT abdomen with IV contrast (if performed)
**Findings**	Gallbladder wall thickening (>3 mm), distension, gallstones or sludge, pericholecystic fluid, sonographic Murphy's sign, hyperemia (on Doppler), complications (empyema, gangrene, perforation, abscess)
**Impression**	No acute cholecystitis / Indeterminate / Acute cholecystitis (uncomplicated or complicated)
**Recommendations**	Surgical consultation / Antibiotics / Follow-up imaging if equivocal

Table 2. Application of the structured reporting template in a synthetic normal case

Section	Content Example
**Clinical Information**	41-year-old female, RUQ pain, no fever, normal WBC
**Imaging Technique**	Abdominal ultrasound
**Findings**	Normal gallbladder wall thickness (2 mm), no gallstones, no distension, no pericholecystic fluid, negative sonographic Murphy's sign
**Impression**	No imaging evidence of acute cholecystitis
**Recommendations**	Consider alternative causes of abdominal pain; no specific imaging follow-up required

Table 3. Application of the structured reporting template in a synthetic indeterminate case

Section	Content Example
**Clinical Information**	55-year-old male, RUQ pain, mild leukocytosis
**Imaging Technique**	Abdominal ultrasound
**Findings**	Mild gallbladder wall thickening (3–4 mm), single gallstone in the neck, equivocal pericholecystic fluid, sonographic Murphy's sign not elicited
**Impression**	Indeterminate; possible early acute cholecystitis
**Recommendations**	Clinical correlation recommended; short-term follow-up ultrasound or CT if symptoms persist or worsen

Table 4. Application of the structured reporting template in a synthetic complicated case

Section	Content Example
**Clinical Information**	68-year-old diabetic male, fever, severe RUQ pain, elevated WBC
**Imaging Technique**	Abdominal ultrasound and contrast-enhanced CT abdomen
**Findings**	Gallbladder markedly distended with thickened, irregular wall, multiple gallstones, large pericholecystic fluid collection, intramural gas consistent with emphysematous cholecystitis
**Impression**	Complicated acute cholecystitis with emphysematous changes and pericholecystic abscess
**Recommendations**	Urgent surgical consultation; IV antibiotics; percutaneous drainage may be considered

S16- Structured Reporting Template for Gallstone Ileus

Table 1. Structured Reporting Template for Gallstone Ileus

Section	Content Example
**Clinical Information**	Age, sex, relevant clinical findings (abdominal pain, nausea/vomiting, distension, history of gallstones or prior cholecystitis)
**Imaging Technique**	Contrast-enhanced CT abdomen and pelvis (preferred); plain abdominal radiograph if available
**Findings**	Evidence of small bowel obstruction (dilated loops, air-fluid levels), ectopic gallstone (location, size), pneumobilia, cholecystoenteric fistula (if seen), complications (perforation, abscess)
**Impression**	No evidence of gallstone ileus / Indeterminate / Gallstone ileus confirmed
**Recommendations**	Surgical consultation; consider enterolithotomy ± fistula repair

Table 2. Application of the structured reporting template in a synthetic normal case

Section	Content Example
**Clinical Information**	59-year-old female, intermittent abdominal pain, no prior gallbladder disease
**Imaging Technique**	Contrast-enhanced CT abdomen and pelvis
**Findings**	Normal caliber small bowel loops, no evidence of obstruction, no ectopic gallstone, gallbladder normal, no pneumobilia
**Impression**	No imaging evidence of gallstone ileus
**Recommendations**	No further imaging required; consider alternative causes of abdominal pain

Table 3. Application of the structured reporting template in a synthetic indeterminate case

Section	Content Example
**Clinical Information**	72-year-old male, nausea, vomiting, abdominal distension, prior history of gallstones
**Imaging Technique**	Contrast-enhanced CT abdomen and pelvis
**Findings**	Mildly dilated small bowel loops with air-fluid levels; a 2 cm calcified density in distal ileum but not clearly intraluminal; equivocal pneumobilia
**Impression**	Indeterminate; possible gallstone ileus (ectopic stone vs vascular calcification)
**Recommendations**	Clinical correlation; repeat CT or enteric contrast study if symptoms progress; surgical evaluation advised

Table 4. Application of the structured reporting template in a synthetic complicated case

Section	Content Example
**Clinical Information**	78-year-old female, severe abdominal pain, vomiting, no bowel movement for 3 days, history of recurrent cholecystitis
**Imaging Technique**	Contrast-enhanced CT abdomen and pelvis
**Findings**	Multiple dilated small bowel loops with transition point at distal ileum, intraluminal gallstone (3.5 cm) at transition site, pneumobilia present, collapsed distal bowel, evidence of cholecysto-duodenal fistula
**Impression**	Gallstone ileus with small bowel obstruction due to impacted gallstone at distal ileum; associated cholecystoenteric fistula
**Recommendations**	Urgent surgical consultation; enterolithotomy indicated; consider fistula management depending on surgical risk

S17- Structured Reporting Template for Mesenteric Ischemia

Table 1. Structured Reporting Template for Mesenteric Ischemia

Section	Content Example
**Clinical Information**	Age, sex, risk factors (atrial fibrillation, atherosclerosis, recent surgery), presenting symptoms (abdominal pain out of proportion, nausea, vomiting, GI bleeding, peritonitis)
**Imaging Technique**	Contrast-enhanced CT angiography of the abdomen and pelvis
**Findings**	Mesenteric vessel patency (SMA, IMA, celiac), thrombus/embolus, bowel wall thickening or thinning, hypoenhancement, pneumatosis intestinalis, portomesenteric venous gas, mesenteric stranding or fluid, perforation
**Impression**	No evidence of mesenteric ischemia / Indeterminate / Mesenteric ischemia confirmed (arterial, venous, or non-occlusive)
**Recommendations**	Urgent surgical consultation / Endovascular or surgical revascularization / Close clinical and imaging follow-up if equivocal

Table 2. Application of the structured reporting template in a synthetic normal case

Section	Content Example
**Clinical Information**	60-year-old male, abdominal pain, atrial fibrillation history
**Imaging Technique**	Contrast-enhanced CT angiography
**Findings**	Patent SMA, IMA, and celiac arteries; no thrombus, embolus, or stenosis; normal bowel wall enhancement; no pneumatosis; no free fluid
**Impression**	No imaging evidence of mesenteric ischemia
**Recommendations**	Consider alternative causes of abdominal pain

Table 3. Application of the structured reporting template in a synthetic indeterminate case

Section	Content Example
**Clinical Information**	72-year-old female, severe abdominal pain, elevated lactate
**Imaging Technique**	Contrast-enhanced CT angiography
**Findings**	Mild mural hypoenhancement in jejunal loops; minimal mesenteric fat stranding; mesenteric vessels appear patent; no pneumatosis or portal venous gas
**Impression**	Indeterminate; possible early mesenteric ischemia
**Recommendations**	Close clinical correlation, repeat imaging if deterioration; surgical consultation recommended

Table 4. Application of the structured reporting template in a synthetic complicated case

Section	Content Example
**Clinical Information**	68-year-old male, atrial fibrillation, sudden severe abdominal pain, hypotension, metabolic acidosis
**Imaging Technique**	Contrast-enhanced CT angiography
**Findings**	Occlusive thrombus at the proximal SMA, extensive non-enhancement of mid and distal small bowel, pneumatosis intestinalis, portomesenteric venous gas, moderate ascites, mesenteric fat stranding
**Impression**	Acute mesenteric ischemia due to SMA thrombosis with transmural bowel infarction
**Recommendations**	Emergent surgical consultation; revascularization vs bowel resection required

S18- Structured Reporting Template for Perforated Hollow Viscus

Table 1. Structured Reporting Template for Perforated Hollow Viscus

Section	Content Example
**Clinical Information**	Age, sex, relevant symptoms (acute abdominal pain, peritonitis, fever, leukocytosis), risk factors (PUD, diverticulitis, trauma, malignancy)
**Imaging Technique**	Contrast-enhanced CT abdomen and pelvis (oral and IV contrast if feasible)
**Findings**	Free intraperitoneal air (site, volume, distribution), free fluid, bowel wall defect, localized thickening, extraluminal oral contrast leak (if given), underlying cause (ulcer, diverticulum, tumor, trauma)
**Impression**	No evidence of perforation / Indeterminate / Perforated hollow viscus (suspected or confirmed site)
**Recommendations**	Surgical consultation; urgent management; follow-up imaging if equivocal

Table 2. Application of the structured reporting template in a synthetic normal case

Section	Content Example
**Clinical Information**	47-year-old male, acute abdominal pain, mild leukocytosis
**Imaging Technique**	Contrast-enhanced CT abdomen and pelvis
**Findings**	No free air, no free fluid, bowel walls normal, no extraluminal contrast, no focal defect
**Impression**	No imaging evidence of perforated hollow viscus
**Recommendations**	Consider alternative causes of abdominal pain

Table 3. Application of the structured reporting template in a synthetic indeterminate case

Section	Content Example
**Clinical Information**	65-year-old female, epigastric pain, history of peptic ulcer disease
**Imaging Technique**	Contrast-enhanced CT abdomen and pelvis
**Findings**	Trace free intraperitoneal air anterior to the liver; no definite bowel wall defect; mild perigastric fat stranding
**Impression**	Indeterminate; possible microperforation (likely gastric/duodenal origin)
**Recommendations**	Correlate with clinical findings and labs; short-term follow-up CT if symptoms persist; surgical consultation advisable

Table 4. Application of the structured reporting template in a synthetic complicated case

Section	Content Example
**Clinical Information**	70-year-old male, severe abdominal pain, fever, rigid abdomen, septic shock
**Imaging Technique**	Contrast-enhanced CT abdomen and pelvis
**Findings**	Large volume free intraperitoneal air in upper abdomen and pelvis, extraluminal oral contrast leaking from anterior gastric wall, diffuse peritonitis with free fluid, thickened adjacent stomach wall
**Impression**	Perforated gastric ulcer with pneumoperitoneum and peritonitis
**Recommendations**	Emergent surgical consultation; urgent operative management required

S19- Structured Reporting Template for Pancreatitis

Table 1. Structured Reporting Template for Pancreatitis

Section	Content Example
**Clinical Information**	Age, sex, relevant symptoms (epigastric pain radiating to back, nausea/vomiting, fever), lab findings (elevated amylase/lipase, leukocytosis)
**Imaging Technique**	Contrast-enhanced CT abdomen and pelvis (pancreatic protocol if available)
**Findings**	Pancreatic size and contour, parenchymal enhancement (normal, heterogeneous, or non-enhancing), peripancreatic fat stranding or fluid, necrosis, pseudocyst, vascular complications (thrombosis, pseudoaneurysm), extrapancreatic involvement (pleural effusion, ascites)
**Impression**	No evidence of pancreatitis / Indeterminate / Acute pancreatitis (with or without complications, classified per Revised Atlanta Classification)
**Recommendations**	Supportive care; clinical follow-up; repeat imaging in severe/complicated cases; surgical or interventional consultation if needed

Table 2. Application of the structured reporting template in a synthetic normal case

Section	Content Example
**Clinical Information**	40-year-old female, epigastric pain, mildly elevated amylase
**Imaging Technique**	Contrast-enhanced CT abdomen and pelvis
**Findings**	Normal pancreatic size and homogeneous enhancement; no peripancreatic stranding or fluid; no necrosis
**Impression**	No imaging evidence of acute pancreatitis
**Recommendations**	Clinical correlation advised; consider non-pancreatic causes of abdominal pain

Table 3. Application of the structured reporting template in a synthetic indeterminate case

Section	Content Example
**Clinical Information**	45-year-old male, epigastric pain radiating to back, elevated lipase
**Imaging Technique**	Contrast-enhanced CT abdomen and pelvis
**Findings**	Mildly enlarged pancreas with subtle peripancreatic fat stranding; enhancement preserved; no necrosis or collections
**Impression**	Indeterminate; findings could represent early acute pancreatitis
**Recommendations**	Clinical correlation recommended; short-term follow-up imaging if symptoms worsen

Table 4. Application of the structured reporting template in a synthetic complicated case

Section	Content Example
**Clinical Information**	56-year-old male, severe epigastric pain, fever, elevated lipase, tachycardia
**Imaging Technique**	Contrast-enhanced CT abdomen and pelvis
**Findings**	Enlarged pancreas with heterogeneous, non-enhancing areas (>30% necrosis); extensive peripancreatic fat stranding and fluid collections; early walled-off necrosis; splenic vein thrombosis with collateral formation; small left pleural effusion
**Impression**	Severe acute necrotizing pancreatitis with peripancreatic collections and vascular complication (splenic vein thrombosis)
**Recommendations**	Intensive care and supportive management; interventional radiology or surgical consultation for drainage if collections progress

S20- Structured Reporting Template for Diverticulitis

Table 1. Structured Reporting Template for Diverticulitis

Section	Content Example
**Clinical Information**	Age, sex, relevant symptoms (LLQ pain, fever, change in bowel habits), lab results (leukocytosis, CRP), history of diverticulosis
**Imaging Technique**	Contrast-enhanced CT abdomen and pelvis
**Findings**	Bowel wall thickening, diverticula, pericolic fat stranding, pericolic fluid, abscess, extraluminal air, fistula, obstruction, Hinchey classification if applicable
**Impression**	No diverticulitis / Indeterminate / Acute diverticulitis (uncomplicated or complicated)
**Recommendations**	Medical management (antibiotics, bowel rest) / Surgical or interventional consultation if complicated / Follow-up imaging if equivocal

Table 2. Application of the structured reporting template in a synthetic normal case

Section	Content Example
**Clinical Information**	52-year-old male, LLQ pain, low-grade fever
**Imaging Technique**	Contrast-enhanced CT abdomen and pelvis
**Findings**	Sigmoid colon with scattered diverticula; no wall thickening, no pericolic fat stranding, no fluid or air
**Impression**	Colonic diverticulosis without evidence of acute diverticulitis
**Recommendations**	No acute intervention required; routine clinical management of diverticulosis

Table 3. Application of the structured reporting template in a synthetic indeterminate case

Section	Content Example
**Clinical Information**	64-year-old female, LLQ pain, leukocytosis
**Imaging Technique**	Contrast-enhanced CT abdomen and pelvis
**Findings**	Mild sigmoid wall thickening with minimal pericolic fat stranding; a few diverticula noted; no abscess or free air
**Impression**	Indeterminate; findings could represent very early diverticulitis vs. nonspecific colitis
**Recommendations**	Clinical correlation recommended; follow-up imaging if symptoms persist or worsen

Table 4. Application of the structured reporting template in a synthetic complicated case

Section	Content Example
**Clinical Information**	70-year-old male, severe LLQ pain, fever, elevated WBC, peritoneal signs
**Imaging Technique**	Contrast-enhanced CT abdomen and pelvis
**Findings**	Marked thickening of sigmoid colon with diverticula, severe pericolic fat stranding, 4 cm pericolic abscess, small extraluminal air bubbles consistent with microperforation, moderate free fluid
**Impression**	Complicated acute sigmoid diverticulitis with pericolic abscess and microperforation (Hinchey II)
**Recommendations**	Urgent surgical or interventional radiology consultation; IV antibiotics; consider percutaneous abscess drainage

S21- Structured Reporting Template for Ovarian Torsion

Table 1. Structured Reporting Template for Ovarian Torsion

Section	Content Example
**Clinical Information**	Age, sex (female), symptoms (acute pelvic pain, nausea/vomiting), relevant history (ovarian cyst, assisted reproduction, prior torsion)
**Imaging Technique**	Pelvic ultrasound with Doppler ± Contrast-enhanced CT/MRI if needed
**Findings**	Ovarian size and morphology, presence of cyst/mass, stromal edema, peripheral follicles, ovarian position (displacement), twisted pedicle ("whirlpool sign"), vascularity (arterial/venous flow), free pelvic fluid
**Impression**	No evidence of torsion / Indeterminate / Ovarian torsion confirmed (specify side)
**Recommendations**	Urgent gynecologic consultation; surgical evaluation

Table 2. Application of the structured reporting template in a synthetic normal case

Section	Content Example
**Clinical Information**	25-year-old female, acute lower abdominal pain, no prior gynecologic disease
**Imaging Technique**	Pelvic ultrasound with Doppler
**Findings**	Normal-sized ovaries with normal morphology and vascular flow; no adnexal mass; no free fluid
**Impression**	No imaging evidence of ovarian torsion
**Recommendations**	Consider non-gynecologic causes of pelvic pain

Table 3. Application of the structured reporting template in a synthetic indeterminate case

Section	Content Example
**Clinical Information**	30-year-old female, acute right lower quadrant pain, history of ovarian cyst
**Imaging Technique**	Pelvic ultrasound with Doppler
**Findings**	Enlarged right ovary (5.5 cm) with peripheral follicles and mild stromal edema; arterial flow preserved, venous flow equivocal; small amount of free pelvic fluid
**Impression**	Indeterminate; partial/intermittent torsion cannot be excluded
**Recommendations**	Close clinical correlation; urgent gynecology consultation; repeat imaging if symptoms persist or worsen

Table 4. Application of the structured reporting template in a synthetic complicated case

Section	Content Example
**Clinical Information**	35-year-old female, severe sudden-onset left lower quadrant pain, nausea/vomiting, leukocytosis
**Imaging Technique**	Pelvic ultrasound with Doppler + CT pelvis
**Findings**	Enlarged left ovary (8 cm) with complex cystic lesion; absent arterial and venous Doppler flow; twisted vascular pedicle ("whirlpool sign"); moderate free pelvic fluid
**Impression**	Left ovarian torsion with underlying complex cyst; vascular compromise confirmed
**Recommendations**	Emergent gynecologic consultation; urgent surgical intervention indicated

S22- Structured Reporting Template for Ectopic Pregnancy

Table 1. Structured Reporting Template for Ectopic Pregnancy

Section	Content Example
**Clinical Information**	Age, sex (female), last menstrual period, β-hCG level, presenting symptoms (pelvic pain, vaginal bleeding, syncope), obstetric/gynecologic history
**Imaging Technique**	Transvaginal ultrasound (preferred) ± Transabdominal ultrasound; MRI/CT if atypical presentation
**Findings**	Presence/absence of intrauterine gestational sac, adnexal mass (size, echogenicity, gestational sac-like structure, yolk sac, fetal pole, cardiac activity), tubal ring sign, free fluid (mild, moderate, massive/hemoperitoneum), rupture signs
**Impression**	No evidence of ectopic pregnancy / Indeterminate / Ectopic pregnancy confirmed (specify location: tubal, cervical, ovarian, abdominal)
**Recommendations**	Gynecology consultation; medical vs surgical management depending on stability and β-hCG level; urgent intervention if rupture suspected

Table 2. Application of the structured reporting template in a synthetic normal case

Section	Content Example
**Clinical Information**	27-year-old female, pelvic pain, β-hCG 2500 IU/L
**Imaging Technique**	Transvaginal ultrasound
**Findings**	Intrauterine gestational sac with yolk sac identified; no adnexal mass; no free fluid
**Impression**	Intrauterine pregnancy; no evidence of ectopic pregnancy
**Recommendations**	Routine obstetric follow-up

Table 3. Application of the structured reporting template in a synthetic indeterminate case

Section	Content Example
**Clinical Information**	30-year-old female, vaginal bleeding, pelvic pain, β-hCG 1800 IU/L
**Imaging Technique**	Transvaginal ultrasound
**Findings**	No intrauterine gestational sac visualized; small right adnexal hypoechoic lesion (1.5 cm) without definitive yolk sac or fetal pole; trace free pelvic fluid
**Impression**	Indeterminate — pregnancy of unknown location (possible early intrauterine vs ectopic)
**Recommendations**	Serial β-hCG follow-up and repeat ultrasound in 48 hours; gynecology consultation

Table 4. Application of the structured reporting template in a synthetic complicated case

Section	Content Example
**Clinical Information**	32-year-old female, acute severe abdominal pain, syncope, β-hCG 3500 IU/L
**Imaging Technique**	Transvaginal ultrasound
**Findings**	No intrauterine gestation; large left adnexal complex mass (3.5 cm) with gestational sac-like structure; moderate-to-large free fluid with echogenic debris (hemoperitoneum)
**Impression**	Ruptured left tubal ectopic pregnancy with hemoperitoneum
**Recommendations**	Emergency gynecology consultation; urgent surgical management required

S23- Structured Reporting Template for Testicular Torsion

Table 1. Structured Reporting Template for Testicular Torsion

Section	Content Example
**Clinical Information**	Age, sex (male), onset/duration of scrotal pain, swelling, nausea/vomiting, prior history of torsion or trauma
**Imaging Technique**	Scrotal ultrasound with color and spectral Doppler
**Findings**	Testicular size and echotexture, parenchymal heterogeneity, vascularity (arterial/venous flow), spermatic cord appearance (whirlpool sign), epididymal changes, scrotal wall thickening, hydrocele, hematocele
**Impression**	No evidence of torsion / Indeterminate (possible intermittent or partial torsion) / Testicular torsion confirmed
**Recommendations**	Urgent urology consultation; surgical detorsion ± orchiopexy; orchiectomy if non-viable

Table 2. Application of the structured reporting template in a synthetic normal case

Section	Content Example
**Clinical Information**	19-year-old male, right scrotal pain after exercise
**Imaging Technique**	Scrotal ultrasound with Doppler
**Findings**	Both testes normal in size and echotexture, symmetric intratesticular arterial and venous flow, no whirlpool sign, no hydrocele
**Impression**	No imaging evidence of testicular torsion
**Recommendations**	Consider alternative causes of scrotal pain (e.g., epididymitis, trauma)

Table 3. Application of the structured reporting template in a synthetic indeterminate case

Section	Content Example
**Clinical Information**	15-year-old male, intermittent left scrotal pain, onset 6 hours ago
**Imaging Technique**	Scrotal ultrasound with Doppler
**Findings**	Left testis slightly enlarged, heterogeneous echotexture; arterial flow present but reduced compared to contralateral side; equivocal "whirlpool sign" of spermatic cord; small reactive hydrocele
**Impression**	Indeterminate; partial or intermittent torsion cannot be excluded
**Recommendations**	Urgent urology consultation; surgical exploration should be strongly considered if clinical suspicion remains high

Table 4. Application of the structured reporting template in a synthetic complicated case

Section	Content Example
**Clinical Information**	21-year-old male, sudden severe right scrotal pain for 10 hours, nausea, vomiting
**Imaging Technique**	Scrotal ultrasound with Doppler
**Findings**	Enlarged right testis with heterogeneous, hypoechoic parenchyma; absent intratesticular arterial and venous flow; twisted spermatic cord with whirlpool sign; moderate reactive hydrocele
**Impression**	Right testicular torsion with absent flow, non-viable appearance
**Recommendations**	Emergency urology consultation; orchiectomy likely required if testis non-viable intraoperatively

S24- Structured Reporting Template for Small Bowel Perforation

Table 1. Structured Reporting Template for Small Bowel Perforation

Section	Content Example
**Clinical Information**	Age, sex, clinical presentation (acute abdominal pain, peritonitis, fever, sepsis, trauma, prior surgery), relevant lab values (WBC, CRP, lactate)
**Imaging Technique**	Contrast-enhanced CT abdomen and pelvis (oral and IV contrast if feasible)
**Findings**	Free intraperitoneal air (location, volume), free fluid, focal bowel wall defect, segmental wall thickening, extraluminal oral contrast leak (if given), localized inflammation, abscess, mesenteric fat stranding, suspected etiology (trauma, obstruction, ischemia, Crohn's disease, tumor)
**Impression**	No evidence of small bowel perforation / Indeterminate / Small bowel perforation confirmed (specify site/etiology)
**Recommendations**	Surgical consultation; urgent operative management if confirmed; follow-up imaging if equivocal

Table 2. Application of the structured reporting template in a synthetic normal case

Section	Content Example
**Clinical Information**	40-year-old male, abdominal pain after blunt trauma, hemodynamically stable
**Imaging Technique**	Contrast-enhanced CT abdomen and pelvis
**Findings**	Normal caliber small bowel loops, intact walls, no free intraperitoneal air or free fluid, no extraluminal contrast
**Impression**	No imaging evidence of small bowel perforation
**Recommendations**	Observation and clinical follow-up as indicated

Table 3. Application of the structured reporting template in a synthetic indeterminate case

Section	Content Example
**Clinical Information**	55-year-old female, abdominal pain, leukocytosis, low-grade fever
**Imaging Technique**	Contrast-enhanced CT abdomen and pelvis
**Findings**	Tiny focus of extraluminal gas adjacent to distal ileum; mild localized mesenteric fat stranding; no definite wall defect; no free fluid
**Impression**	Indeterminate — possible small contained perforation vs benign intraperitoneal gas (e.g., post-procedure)
**Recommendations**	Clinical correlation and close monitoring; repeat CT if symptoms worsen

Table 4. Application of the structured reporting template in a synthetic complicated case

Section	Content Example
**Clinical Information**	70-year-old male, acute severe abdominal pain, peritonitis, septic shock
**Imaging Technique**	Contrast-enhanced CT abdomen and pelvis
**Findings**	Large volume free intraperitoneal air diffusely distributed; thickened ischemic segment of jejunum with focal wall defect; moderate free fluid with inflammatory changes; mesenteric stranding
**Impression**	Small bowel perforation due to ischemic segment (jejunal origin) with diffuse peritonitis
**Recommendations**	Emergent surgical consultation; urgent operative management required

S25- Structured Reporting Template for Peritonitis

Table 1. Structured Reporting Template for Peritonitis

Section	Content Example
**Clinical Information**	Age, sex, presenting symptoms (diffuse abdominal pain, fever, rigidity, sepsis), relevant history (trauma, recent surgery, GI perforation, PID, cirrhosis with SBP)
**Imaging Technique**	Contrast-enhanced CT abdomen and pelvis (IV ± oral contrast)
**Findings**	Free fluid (amount, distribution, character), free intraperitoneal air, bowel wall abnormalities, peritoneal thickening/enhancement, abscess formation, mesenteric fat stranding, underlying source (perforation, appendicitis, diverticulitis, trauma)
**Impression**	No peritonitis / Indeterminate / Peritonitis confirmed (secondary or primary, localized or generalized)
**Recommendations**	Surgical consultation if secondary peritonitis; targeted therapy (e.g., antibiotics, drainage); follow-up imaging if equivocal

Table 2. Application of the structured reporting template in a synthetic normal case

Section	Content Example
**Clinical Information**	44-year-old female, diffuse abdominal pain, mild leukocytosis
**Imaging Technique**	Contrast-enhanced CT abdomen and pelvis
**Findings**	No free fluid, no free air, normal bowel walls, no peritoneal thickening
**Impression**	No imaging evidence of peritonitis
**Recommendations**	Consider alternative causes of abdominal pain

Table 3. Application of the structured reporting template in a synthetic indeterminate case

Section	Content Example
**Clinical Information**	61-year-old male, abdominal distension, moderate leukocytosis, cirrhosis
**Imaging Technique**	Contrast-enhanced CT abdomen and pelvis
**Findings**	Moderate ascites; minimal peritoneal enhancement; no obvious perforation, abscess, or free air
**Impression**	Indeterminate — possible spontaneous bacterial peritonitis vs sterile ascites
**Recommendations**	Clinical correlation; paracentesis for analysis; follow-up imaging if symptoms persist

Table 4. Application of the structured reporting template in a synthetic complicated case

Section	Content Example
**Clinical Information**	68-year-old male, severe diffuse abdominal pain, fever, septic shock
**Imaging Technique**	Contrast-enhanced CT abdomen and pelvis
**Findings**	Large volume free intraperitoneal air and fluid, diffuse peritoneal thickening and enhancement, multiple abscess pockets along paracolic gutters, thickened sigmoid colon with perforation site identified
**Impression**	Secondary generalized peritonitis due to perforated sigmoid diverticulitis with abscess formation
**Recommendations**	Emergent surgical consultation; broad-spectrum antibiotics; percutaneous drainage may be required for collections

S26- Structured Reporting Template for Pulmonary Edema

Table 1. Structured Reporting Template for Pulmonary Edema

Section	Content Example
**Clinical Information**	Age, sex, relevant symptoms (dyspnea, orthopnea, cough, chest pain), past history (heart failure, renal failure, ARDS risk, sepsis)
**Imaging Technique**	Chest radiograph ± Chest CT (contrast-enhanced if evaluating for PE or ARDS)
**Findings**	Heart size (normal/enlarged), pulmonary vascular congestion, interstitial edema (Kerley B lines, peribronchial cuffing), alveolar edema (ground-glass opacities, consolidations, perihilar "bat-wing" pattern), pleural effusions, air bronchograms, septal thickening; distribution (central vs peripheral, symmetrical vs asymmetrical); differentiation between cardiogenic vs non-cardiogenic pattern
**Impression**	No pulmonary edema / Indeterminate / Pulmonary edema (cardiogenic or non-cardiogenic)
**Recommendations**	Clinical correlation (cardiac vs non-cardiac causes); echocardiography if cardiogenic suspected; ICU-level supportive management in severe cases

Table 2. Application of the structured reporting template in a synthetic normal case

Section	Content Example
**Clinical Information**	55-year-old male, mild dyspnea, history of hypertension
**Imaging Technique**	Chest radiograph (PA and lateral)
**Findings**	Normal cardiac size, clear lung fields, no septal lines, no pleural effusion
**Impression**	No imaging evidence of pulmonary edema
**Recommendations**	Consider non-cardiopulmonary causes of dyspnea

Table 3. Application of the structured reporting template in a synthetic indeterminate case

Section	Content Example
**Clinical Information**	60-year-old female, acute dyspnea, renal failure on dialysis
**Imaging Technique**	Chest radiograph
**Findings**	Mild cardiomegaly, minimal bilateral perihilar haziness, equivocal Kerley B lines, no definite pleural effusion
**Impression**	Indeterminate — early interstitial edema cannot be excluded
**Recommendations**	Correlate with clinical and laboratory findings (BNP, renal function); repeat chest imaging if symptoms progress

Table 4. Application of the structured reporting template in a synthetic complicated case

Section	Content Example
**Clinical Information**	68-year-old male, acute severe dyspnea, orthopnea, history of ischemic cardiomyopathy
**Imaging Technique**	Chest radiograph and contrast-enhanced chest CT
**Findings**	Cardiomegaly, pulmonary venous congestion, diffuse bilateral ground-glass opacities with perihilar "bat-wing" pattern, Kerley B lines, bilateral pleural effusions, air bronchograms present
**Impression**	Severe cardiogenic pulmonary edema with bilateral pleural effusions
**Recommendations**	Urgent cardiology consultation; ICU monitoring and supportive therapy

S27- Structured Reporting Template for Pneumonia

Table 1. Structured Reporting Template for Pneumonia

Section	Content Example
**Clinical Information**	Age, sex, symptoms (fever, cough, dyspnea, pleuritic pain), risk factors (smoking, aspiration risk, immunosuppression), lab results (WBC, CRP, procalcitonin)
**Imaging Technique**	Chest radiograph (PA/Lateral) ± Contrast-enhanced chest CT if complications/atypical features suspected
**Findings**	Distribution (lobar/segmental/multifocal/diffuse), pattern (consolidation, ground-glass opacities, centrilobular nodules/tree-in-bud), air bronchograms, bronchial wall thickening, atelectasis, pleural effusion/empyema, cavitation/abscess, pneumatocele, lymphadenopathy; laterality and lobar involvement; comparison with priors
**Impression**	No pneumonia / Indeterminate / Findings consistent with pneumonia (specify likely etiology if suggestive: typical bacterial, aspiration, atypical/viral), side/lobes involved, severity; list complications if present
**Recommendations**	Correlate clinically and microbiologically; antibiotics per guidelines; follow-up imaging for resolution (e.g., 6–8 weeks in older smokers or if lobar collapse/concern for malignancy); CT if complicated/atypical course

Table 2. Application in a synthetic normal case

Section	Content Example
**Clinical Information**	45-year-old female, cough and low-grade fever, normal oxygen saturation
**Imaging Technique**	Chest radiograph (PA/Lateral)
**Findings**	Clear lungs bilaterally; no consolidation, no ground-glass opacities; cardiac and mediastinal contours normal; no pleural effusion
**Impression**	No radiographic evidence of pneumonia
**Recommendations**	Consider non-pulmonary or early/non-radiographic infection; clinical follow-up as needed

Table 3. Application in a synthetic indeterminate case

Section	Content Example
**Clinical Information**	67-year-old male, fever and productive cough, smoker
**Imaging Technique**	Chest radiograph
**Findings**	Subtle patchy opacity at the right lower zone, partially silhouetting the right hemidiaphragm; mild bronchitic markings; no effusion
**Impression**	Indeterminate — early pneumonia vs subsegmental atelectasis
**Recommendations**	Clinical and lab correlation; consider repeat radiograph in 24–48 hours or CT if symptoms severe/persisting

Table 4. Application in a synthetic complicated case

Section	Content Example
**Clinical Information**	72-year-old female, high fever, pleuritic chest pain, hypoxia
**Imaging Technique**	Chest radiograph and contrast-enhanced chest CT
**Findings**	Dense right middle and lower lobe consolidation with air bronchograms; moderate right pleural effusion with split-pleura sign on CT consistent with empyema; small cavitating focus in posterior RLL suggesting necrotizing pneumonia
**Impression**	Severe right-sided bacterial pneumonia (middle and lower lobes) complicated by empyema and focal necrotizing change

Section	Content Example
**Recommendations**	Urgent antibiotics; respiratory/ID and interventional consultation for pleural drainage; follow-up imaging to document resolution after therapy

S28- Structured Reporting Template for COVID-19 Pneumonia

Table 1. Structured Reporting Template for COVID-19 Pneumonia

Section	Content Example
**Clinical Information**	Age, sex, presenting symptoms (fever, cough, dyspnea, hypoxia), known/suspected COVID-19 exposure, PCR status
**Imaging Technique**	Chest radiograph ± Chest CT (non-contrast preferred unless other indications)
**Findings**	Ground-glass opacities (GGO), consolidation, distribution (bilateral/unilateral, peripheral vs central, basal vs diffuse), crazy-paving pattern, vascular thickening, pleural effusion (rare), lymphadenopathy (rare), complications (ARDS, pneumothorax, bacterial superinfection)
**Impression**	No pneumonia / Indeterminate for COVID-19 / Findings typical of COVID-19 pneumonia / Alternative diagnosis favored
**Recommendations**	Correlate with PCR/clinical status; isolation precautions; follow-up imaging if worsening; ICU management if severe

Table 2. Application in a synthetic normal case

Section	Content Example
**Clinical Information**	35-year-old female, fever, cough, COVID-19 contact, PCR negative
**Imaging Technique**	Chest CT (non-contrast)
**Findings**	Clear lung parenchyma; no ground-glass opacities or consolidation; no pleural effusion
**Impression**	No imaging evidence of COVID-19 pneumonia
**Recommendations**	Continue clinical observation and repeat PCR testing if suspicion remains high

Table 3. Application in a synthetic indeterminate case

Section	Content Example
**Clinical Information**	50-year-old male, fever, cough, PCR pending
**Imaging Technique**	Chest CT
**Findings**	Single patchy ground-glass opacity in the right lower lobe; no bilateral or peripheral distribution; no consolidation
**Impression**	Indeterminate for COVID-19 pneumonia (could represent viral pneumonia or early atypical bacterial infection)
**Recommendations**	Correlate with PCR and clinical course; repeat imaging if symptoms progress

Table 4. Application in a synthetic complicated case

Section	Content Example
**Clinical Information**	68-year-old male, hypoxia, confirmed COVID-19 positive, ICU admission
**Imaging Technique**	Chest CT (non-contrast)
**Findings**	Diffuse bilateral ground-glass opacities with superimposed consolidation ("crazy paving" pattern), peripheral and basal predominance; small right pneumothorax; no pleural effusion
**Impression**	Severe COVID-19 pneumonia with diffuse bilateral lung involvement, complicated by pneumothorax
**Recommendations**	Urgent ICU management; ventilatory support; monitor for ARDS and superinfection

S29- Structured Reporting Template for Pneumomediastinum

Table 1. Structured Reporting Template for Pneumomediastinum

Section	Content Example
**Clinical Information**	Age, sex, presenting symptoms (chest pain, dyspnea, subcutaneous emphysema, voice changes), risk factors (trauma, vomiting/Boerhaave, asthma exacerbation, mechanical ventilation, COVID-19)
**Imaging Technique**	Chest radiograph ± Contrast-enhanced CT chest (gold standard)
**Findings**	Presence, volume, and distribution of mediastinal air (anterior, posterior, superior, inferior compartments); subcutaneous emphysema; extension into pericardium (pneumopericardium); associated findings (esophageal perforation, tracheobronchial injury, lung disease, pneumothorax, pneumoperitoneum)
**Impression**	No pneumomediastinum / Indeterminate / Pneumomediastinum confirmed (specify cause if suspected)
**Recommendations**	Clinical correlation; conservative management vs urgent surgical evaluation depending on suspected etiology (e.g., perforation)

Table 2. Application in a synthetic normal case

Section	Content Example
**Clinical Information**	30-year-old male, chest pain after exertion
**Imaging Technique**	Chest radiograph and CT
**Findings**	Normal lung fields, normal mediastinal contours; no free mediastinal air; no subcutaneous emphysema
**Impression**	No imaging evidence of pneumomediastinum
**Recommendations**	Consider alternative causes of chest pain

Table 3. Application in a synthetic indeterminate case

Section	Content Example
**Clinical Information**	45-year-old female, chest tightness after endoscopy
**Imaging Technique**	Chest radiograph
**Findings**	Equivocal lucency along the left mediastinal border; no definite free air on lateral view; no subcutaneous emphysema
**Impression**	Indeterminate — questionable pneumomediastinum vs artifact
**Recommendations**	Contrast-enhanced CT chest/esophagogram recommended to rule out pneumomediastinum and esophageal injury

Table 4. Application in a synthetic complicated case

Section	Content Example
**Clinical Information**	62-year-old male, severe chest pain and dyspnea after forceful vomiting, hypotension
**Imaging Technique**	Contrast-enhanced CT chest/abdomen
**Findings**	Extensive free mediastinal air tracking into the neck and pericardium; associated left hydropneumothorax; focal extraluminal contrast leak from distal esophagus consistent with Boerhaave syndrome
**Impression**	Pneumomediastinum with esophageal perforation (Boerhaave syndrome), complicated by hydropneumothorax
**Recommendations**	Emergent surgical consultation; broad-spectrum antibiotics; ICU management

S30- Structured Reporting Template for Myocardial Infarction (Complications)

Table 1. Structured Reporting Template for Myocardial Infarction (Complications)

Section	Content Example
**Clinical Information**	Age, sex, history of acute MI, chest pain, hypotension, arrhythmia, dyspnea
**Imaging Technique**	Echocardiography (initial); Cardiac CT/MRI for detailed assessment; Coronary angiography if intervention planned
**Findings**	LV wall motion abnormalities, thinning, aneurysm/pseudoaneurysm formation, interventricular septal defect (VSD), papillary muscle rupture, mitral regurgitation, LV thrombus, pericardial effusion ± tamponade, myocardial rupture
**Impression**	No complication / Indeterminate / Complication of MI confirmed (specify type)
**Recommendations**	Urgent cardiology/cardiothoracic surgical consultation; hemodynamic support; surgical or interventional management if required

Table 2. Application in a synthetic normal post-MI case

Section	Content Example
**Clinical Information**	58-year-old male, inferior MI 1 week ago, stable
**Imaging Technique**	Echocardiography
**Findings**	Regional wall motion abnormality (inferior hypokinesis), preserved LV wall thickness, no aneurysm, no thrombus, no pericardial effusion
**Impression**	No imaging evidence of mechanical complication of MI
**Recommendations**	Continue routine post-MI management and follow-up

Table 3. Application in a synthetic indeterminate case

Section	Content Example
**Clinical Information**	65-year-old female, anterior MI 10 days ago, new systolic murmur
**Imaging Technique**	Echocardiography
**Findings**	Hypokinetic anterior wall; small area of septal thinning with turbulent color Doppler flow suggestive of possible VSD; findings not conclusive
**Impression**	Indeterminate — possible post-MI ventricular septal rupture
**Recommendations**	Recommend urgent cardiac MRI or contrast echocardiography; cardiology consultation

Table 4. Application in a synthetic complicated case

Section	Content Example
**Clinical Information**	72-year-old male, large anterior MI 5 days ago, sudden hypotension and syncope
**Imaging Technique**	Echocardiography and Cardiac CT
**Findings**	Large pseudoaneurysm arising from the LV anterior wall with narrow neck, contained rupture; large pericardial effusion with signs of tamponade; no LV thrombus visualized
**Impression**	Complicated post-MI pseudoaneurysm with contained rupture and hemopericardium causing tamponade
**Recommendations**	Emergent cardiothoracic surgery consultation; surgical repair required

S31- Structured Reporting Template for Deep Vein Thrombosis (DVT)

Table 1. Structured Reporting Template for Deep Vein Thrombosis (DVT)

Section	Content Example
**Clinical Information**	Age, sex, symptoms (limb swelling, pain, redness), risk factors (immobilization, malignancy, recent surgery, pregnancy, prior DVT/PE)
**Imaging Technique**	Duplex Doppler ultrasound of lower extremity veins (common femoral → calf veins ± iliac veins)
**Findings**	Vein compressibility, intraluminal echogenic material, venous diameter, color Doppler flow (present/absent), spectral waveform analysis, extent of thrombus (distal/proximal, occlusive/non-occlusive), collateral formation
**Impression**	No DVT / Indeterminate / DVT present (specify location, extent, occlusion status)
**Recommendations**	Anticoagulation therapy if confirmed; vascular medicine or interventional radiology consultation if extensive/proximal; follow-up ultrasound for propagation or recanalization

Table 2. Application in a synthetic normal case

Section	Content Example
**Clinical Information**	40-year-old female, right calf pain, recent long flight
**Imaging Technique**	Duplex Doppler ultrasound of right lower extremity
**Findings**	Common femoral, superficial femoral, popliteal, and calf veins all fully compressible; normal intraluminal flow with color Doppler; no thrombus seen
**Impression**	No imaging evidence of DVT in the right lower extremity
**Recommendations**	Consider musculoskeletal causes of calf pain; clinical follow-up if symptoms persist

Table 3. Application in a synthetic indeterminate case

Section	Content Example
**Clinical Information**	67-year-old male, left leg swelling and pain, history of malignancy
**Imaging Technique**	Duplex Doppler ultrasound of left lower extremity
**Findings**	Non-compressible left peroneal vein with questionable intraluminal echogenic focus; flow equivocal due to technical limitations; more proximal veins appear patent
**Impression**	Indeterminate — possible isolated calf DVT; not definitively proven
**Recommendations**	Short-term follow-up ultrasound in 5–7 days or consider venography/CT venography if high clinical suspicion

Table 4. Application in a synthetic complicated case

Section	Content Example
**Clinical Information**	72-year-old female, severe left leg swelling, shortness of breath, tachycardia
**Imaging Technique**	Duplex Doppler ultrasound of left lower extremity
**Findings**	Non-compressible common femoral, superficial femoral, and popliteal veins filled with echogenic thrombus; absent venous flow on Doppler; extensive collateral formation
**Impression**	Extensive occlusive DVT of the left lower extremity (iliofemoral DVT)
**Recommendations**	Urgent initiation of anticoagulation; vascular/interventional consultation for thrombolysis or thrombectomy; evaluate for pulmonary embolism given clinical symptoms

S32- Structured Reporting Template for Seizure-Related Stroke Mimic

Table 1. Structured Reporting Template for Seizure-Related Stroke Mimic

Section	Content Example
**Clinical Information**	Age, sex, acute neurological deficit (weakness, speech disturbance, visual symptoms), seizure activity (postictal state, Todd's paralysis), risk factors (epilepsy history, recent seizure, alcohol withdrawal, metabolic derangements)
**Imaging Technique**	Non-contrast brain CT ± CT angiography ± MRI (DWI, FLAIR, GRE/SWI, perfusion)
**Findings**	**CT**: No acute hemorrhage or territorial infarction.<br>**MRI**: Transient cortical/subcortical T2/FLAIR hyperintensity, DWI hyperintensity without ADC restriction, perfusion may show hyperperfusion.<br>**Vessels**: No large vessel occlusion.<br>**Ancillary**: Possible mild cortical swelling, gyral enhancement in subacute phase.
**Impression**	No evidence of acute ischemic stroke; findings suggest seizure-related changes (Todd's paralysis or peri-ictal changes)
**Recommendations**	Clinical correlation with seizure history and EEG; short-interval MRI follow-up if deficits persist; avoid unnecessary thrombolysis if mimic confirmed

Table 2. Application in a synthetic normal case

Section	Content Example
**Clinical Information**	32-year-old male with left arm weakness following a generalized tonic–clonic seizure
**Imaging Technique**	Non-contrast CT brain
**Findings**	No acute hemorrhage, infarction, or mass effect. Gray-white matter differentiation preserved.
**Impression**	No imaging evidence of acute stroke. Clinical features likely represent postictal (Todd's) paralysis.
**Recommendations**	Supportive management, EEG correlation; symptoms expected to resolve within hours.

Table 3. Application in a synthetic indeterminate case

Section	Content Example
**Clinical Information**	58-year-old female, acute right-sided weakness after focal seizure; persistent deficit for >6 hours
**Imaging Technique**	MRI brain with DWI/FLAIR
**Findings**	Cortical hyperintensity on DWI in left parietal lobe, but no ADC restriction; FLAIR hyperintensity with mild cortical swelling; no vascular occlusion on MRA.
**Impression**	Findings suggest seizure-related transient changes rather than acute infarct; however, stroke cannot be fully excluded.
**Recommendations**	Repeat MRI in 24–48 hours for evolution; close clinical/EEG correlation.

Table 4. Application in a synthetic complicated case

Section	Content Example
**Clinical Information**	70-year-old male, known epilepsy, sudden aphasia and hemiparesis after seizure; initial suspicion for stroke
**Imaging Technique**	MRI brain with DWI, ADC, perfusion
**Findings**	Left temporal and parietal cortical hyperintensity on FLAIR and DWI without diffusion restriction (ADC preserved). Perfusion shows regional hyperperfusion. No large vessel occlusion.
**Impression**	Imaging features favor seizure-related peri-ictal changes mimicking acute ischemic stroke. No acute infarction identified.
**Recommendations**	Withhold thrombolysis; manage as seizure with EEG and antiepileptic adjustment. Short-term follow-up imaging to confirm resolution.

S33- Structured Reporting Template for Spinal Trauma

Table 1. Structured Reporting Template for Spinal Trauma

Section	Content Example
**Clinical Information**	Age, sex, mechanism of trauma (fall, traffic accident, sports injury), symptoms (pain, neurological deficit, numbness, weakness, sphincter disturbance)
**Imaging Technique**	CT (bone window, multiplanar reconstruction) ± MRI (cord signal, ligamentous injury, soft tissue, hematoma)
**Vertebral Alignment**	Normal / malalignment / listhesis (antero-, retro-, lateral), scoliosis, kyphosis
**Vertebral Body**	Normal height / compression / burst fracture / fracture–dislocation
**Posterior Elements**	Intact / fractures of pedicles, laminae, spinous processes, facets
**Intervertebral Discs & Endplates**	Intact / disruption / disc herniation
**Ligaments (MRI)**	Intact / injury to ALL, PLL, ligamentum flavum, interspinous, supraspinous
**Spinal Canal & Cord**	Canal stenosis, retropulsed fragments, cord compression, intramedullary signal (edema, contusion, hemorrhage), epidural hematoma
**Other Findings**	Paraspinal soft tissue swelling, hematoma, vascular injury
**Impression**	Stable vs unstable fracture, presence of spinal cord injury
**Recommendations**	Urgent neurosurgical/orthopedic consultation if unstable or neurological deficit; MRI if only CT performed and suspicion of cord/ligament injury

Table 2. Application in a synthetic normal case

Section	Content Example
**Clinical Information**	25-year-old male after minor motor vehicle accident, presenting with mild neck pain
**Imaging Technique**	Cervical CT (multiplanar reconstructions)
**Findings**	Normal vertebral alignment and body height. No fractures. Intervertebral discs and posterior elements intact. No canal narrowing or cord compression.
**Impression**	No CT evidence of acute spinal trauma.
**Recommendations**	Clinical follow-up; MRI if persistent neurological symptoms.

Table 3. Application in a synthetic indeterminate case

Section	Content Example
**Clinical Information**	47-year-old female after fall from height, presenting with thoracolumbar pain, no neurological deficit
**Imaging Technique**	Thoracolumbar CT
**Findings**	Mild wedge compression fracture at T12 with 15% anterior height loss, no retropulsion. Posterior elements intact. Alignment preserved.
**Impression**	Stable T12 compression fracture; no canal compromise.
**Recommendations**	Conservative management; MRI if pain disproportionate to CT findings or if ligamentous injury suspected.

Table 4. Application in a synthetic complicated case

Section	Content Example
**Clinical Information**	60-year-old male after high-speed car accident, presenting with paraplegia
**Imaging Technique**	Thoracic CT and MRI
**Findings**	T7 burst fracture with >50% loss of vertebral body height and retropulsed bony fragments causing severe canal stenosis. Posterior element fractures with dislocation. MRI shows cord compression with

Section	Content Example
	intramedullary T2 hyperintensity and small focus of GRE hypointensity (cord hemorrhage). Disruption of posterior ligamentous complex.
**Impression**	Unstable T7 burst fracture with spinal cord compression and hemorrhagic contusion.
**Recommendations**	Emergency neurosurgical intervention. MRI follow-up if required for surgical planning.

S34- Structured Reporting Template for Cauda Equina Syndrome

Table 1. Structured Reporting Template for Cauda Equina Syndrome

Section	Content Example
**Clinical Information**	Age, sex; red flags: bilateral sciatica, **urinary retention/incontinence**, reduced anal tone, saddle anesthesia, sexual dysfunction; onset and progression; recent infection, trauma, surgery, anticoagulation
**Imaging Technique**	**MRI lumbar spine**: sagittal/axial T1, T2, STIR; add post-contrast if infection/tumor suspected. (CT myelography if MRI contraindicated.)
**Findings**	Levels involved; **disc pathology** (bulge/protrusion/extrusion/sequestration), size (AP×TR×CC), location (central/para), migration; canal diameter/area; **thecal sac compression**, CSF effacement; **nerve root crowding/clumping**; conus level/signal; **epidural collection** (abscess/hematoma), enhancement pattern; **facet synovial cyst**, spondylolisthesis, fracture; paraspinal/osseous changes; ancillary: markedly distended bladder (if imaged).
**Impression**	No CES by imaging / Indeterminate (narrowing w/o definitive root crowding) / **CES due to**: (e.g., massive central L4–5 extrusion / epidural abscess / hematoma). Include severity (mild/moderate/severe thecal sac compression).
**Recommendations**	If CES confirmed/suspected: **emergent neurosurgical consultation**. If infection suspected: add IV antibiotics and urgent decompression. If indeterminate: correlate with neuro exam and **post-void residual (PVR)**; short-interval reassessment or repeat MRI if deficits progress.

Table 2. Application in a synthetic normal case

Section	Content Example
**Clinical Information**	38-year-old male, acute low-back pain, unilateral sciatica; no urinary symptoms; normal anal tone
**Imaging Technique**	MRI lumbar spine (T1/T2/STIR)
**Findings**	Mild L4–5 annular bulge; **no central canal stenosis**, CSF around cauda equina preserved; **no root crowding or clumping**; conus ends at L1, normal signal; no mass/collection
**Impression**	**No imaging evidence of CES.** Mild degenerative changes only.
**Recommendations**	Clinical management for mechanical back pain/radiculopathy; return precautions for red flags.

Table 3. Application in a synthetic indeterminate case

Section	Content Example
**Clinical Information**	54-year-old female, new bilateral sciatica, vague saddle paresthesia; **PVR 120 mL**
**Imaging Technique**	MRI lumbar spine (non-contrast)
**Findings**	Broad-based **central L4–5 protrusion** narrowing canal; **ventral CSF effacement** but **dorsal CSF preserved**; no definite root clumping; conus normal; no collection
**Impression**	**Indeterminate for CES** — significant central stenosis without definitive cauda equina crowding. **Impending CES cannot be excluded** given symptoms.
**Recommendations**	**Urgent** neurological exam and bladder monitoring; short-interval re-evaluation; consider repeat MRI or expedited decompression if deficits/retention progress.

Table 4. Application in a synthetic complicated case

Section	Content Example
**Clinical Information**	62-year-old male, sudden **urinary retention (800 mL)**, saddle anesthesia, reduced anal tone, bilateral leg weakness
**Imaging Technique**	MRI lumbar spine
**Findings**	**Massive central L5–S1 sequestered disc fragment** (1.8×1.5×1.2 cm) with **near-complete CSF effacement** and **severe cauda equina root crowding**; subtle superior migration behind L5; no abscess/hematoma; conus normal
**Impression**	**Cauda equina syndrome due to large L5–S1 sequestrated disc** with severe thecal sac compression.

Section	Content Example
**Recommendations**	**Emergent neurosurgical decompression**; perioperative bladder management; postoperative MRI if indicated.

S35- Structured Reporting Template for Spinal Epidural Abscess

Table 1. Structured Reporting Template for Spinal Epidural Abscess

Section	Content Example
**Clinical Information**	Age, sex; risk factors (diabetes, IV drug use, immunosuppression, recent spinal intervention, bacteremia); symptoms: fever, back pain, radiculopathy, neurological deficits; onset and progression.
**Imaging Technique**	MRI spine (sagittal/axial T1, T2, STIR; post-contrast mandatory); whole-spine coverage if clinically indicated. (CT only if MRI contraindicated.)
**Findings**	Level(s) involved; epidural collection location (anterior/posterior, dorsal/ventral, circumferential); signal characteristics (T1/T2/STIR); enhancement pattern (rim-enhancing abscess vs. phlegmon); degree of thecal sac and cord/cauda equina compression; nerve root involvement; extension (paraspinal, facet, vertebral body, discitis/osteomyelitis); ancillary: prevertebral/soft tissue swelling, skip lesions.
**Impression**	No evidence of epidural abscess / Indeterminate (enhancement without clear collection) / Epidural abscess confirmed at [level], with [mild/moderate/severe] compression.
**Recommendations**	If abscess confirmed/suspected: emergent neurosurgical consultation + broad-spectrum IV antibiotics. If indeterminate: close clinical and laboratory correlation (WBC, CRP, blood cultures); repeat MRI in short interval. If concomitant osteomyelitis/discitis: multidisciplinary approach with ID and spine surgery.

Table 2. Application in a synthetic normal case

Section	Content Example
**Clinical Information**	41-year-old male, acute low-back pain, no fever, no neurological deficit, no history of infection or immunosuppression
**Imaging Technique**	MRI lumbar spine (T1/T2/STIR with contrast)
**Findings**	No epidural collection; epidural fat preserved; normal thecal sac; no abnormal enhancement; vertebral bodies and discs intact
**Impression**	No imaging evidence of spinal epidural abscess.
**Recommendations**	Clinical management for mechanical back pain; no infectious findings.

Table 3. Application in a synthetic indeterminate case

Section	Content Example
**Clinical Information**	55-year-old diabetic female with fever and worsening back pain; no focal neurological deficit
**Imaging Technique**	MRI lumbar spine (with contrast)
**Findings**	Dorsal epidural soft tissue at L3–L4 showing intermediate T1, hyperintense T2, and patchy enhancement; no definite fluid collection; mild thecal sac compression; no root clumping; vertebral endplates normal
**Impression**	Indeterminate: enhancing epidural phlegmon at L3–L4 without definite rim-enhancing abscess. Early epidural abscess cannot be excluded.
**Recommendations**	Urgent infectious workup (blood cultures, CRP, WBC); close clinical monitoring; repeat MRI in 48–72h or earlier if neurological deficits develop.

Table 4. Application in a synthetic complicated case

Section	Content Example
**Clinical Information**	60-year-old male, IV drug user, presenting with fever, severe back pain, bilateral leg weakness, urinary retention
**Imaging Technique**	MRI thoracolumbar spine (with contrast)
**Findings**	Large rim-enhancing posterior epidural abscess from T11–L2, measuring 3.0 × 1.2 × 5.0 cm; marked dorsal epidural compression with near-complete effacement of CSF and severe cauda equina root crowding; associated L1 vertebral body marrow edema and enhancement (early osteomyelitis); paraspinal extension into left psoas
**Impression**	Extensive spinal epidural abscess T11–L2 with severe cauda equina compression and associated vertebral osteomyelitis.

Section	Content Example
**Recommendations**	Emergent neurosurgical decompression and drainage; initiate broad-spectrum IV antibiotics; ID consultation; follow-up MRI to assess treatment response.

S36- Structured Reporting Template for Subdural Hematoma

Table 1. Structured Reporting Template for Subdural Hematoma

Section	Content Example
**Clinical Information**	Age, sex; history of trauma/fall; anticoagulation use; headache, vomiting, altered mental status, seizures, focal deficits, GCS; acute vs chronic symptoms; prior neurosurgery
**Imaging Technique**	CT head without contrast (first-line); MRI brain if CT equivocal or for subacute/chronic SDH (T1, T2, FLAIR, GRE/SWI, post-contrast if membrane/collection suspected)
**Findings**	Location (right/left/bilateral, convexity, interhemispheric, tentorial, posterior fossa); hematoma thickness (mm), length (AP × CC); density/signal (acute: hyperdense, subacute: iso, chronic: hypo on CT); mass effect (midline shift mm, sulcal effacement, ventricular compression); acute on chronic layering; membranes/septations; associated skull fracture; parenchymal contusion/ischemia; extra lesions (epidural, SAH)
**Impression**	No SDH / Acute SDH / Subacute SDH / Chronic SDH / Mixed-density SDH. State side, size, and significant mass effect (e.g., >5 mm midline shift, effaced basal cisterns).
**Recommendations**	If large SDH with mass effect: urgent neurosurgical evaluation for evacuation. If small/stable: clinical + imaging follow-up. If anticoagulated: reverse coagulopathy. MRI if atypical or membrane suspected.

Table 2. Application in a synthetic normal case

Section	Content Example
**Clinical Information**	45-year-old male, mild head trauma, headache only, GCS 15
**Imaging Technique**	CT brain without contrast
**Findings**	No extra-axial collection; sulci and ventricles preserved; no midline shift; no fracture
**Impression**	No imaging evidence of subdural hematoma.
**Recommendations**	Symptomatic management; discharge with return precautions if neurologically stable.

Table 3. Application in a synthetic indeterminate case

Section	Content Example
**Clinical Information**	70-year-old female, on warfarin, fall 3 days ago, persistent headache, mild confusion
**Imaging Technique**	CT brain without contrast
**Findings**	Left frontoparietal convexity crescentic collection, 5 mm thick, hypodense relative to brain (chronic/subacute); no midline shift; sulci preserved; no fracture
**Impression**	Left chronic subdural hematoma, small, without significant mass effect.
**Recommendations**	Clinical correlation; correct coagulopathy; neurosurgical follow-up; repeat CT in 1–2 weeks or sooner if symptoms progress.

Table 4. Application in a synthetic complicated case

Section	Content Example
**Clinical Information**	68-year-old male, fall, decreased consciousness (GCS 9), right-sided weakness, on aspirin
**Imaging Technique**	CT brain without contrast
**Findings**	Large acute right frontotemporoparietal crescentic hyperdense subdural hematoma measuring up to 18 mm thickness, causing 12 mm leftward midline shift, complete effacement of right lateral ventricle, compression of basal cisterns. Small contralateral chronic hypodense subdural collection. Associated right temporal bone fracture.
**Impression**	Large acute right subdural hematoma with severe mass effect and midline shift. Additional contralateral chronic collection.
**Recommendations**	Emergent neurosurgical evacuation; reverse platelet dysfunction; ICU admission and intracranial pressure monitoring.

S37- Structured Reporting Template for Epidural Hematoma

Table 1. Structured Reporting Template for

Section	Content Example
**Clinical Information**	Age, sex; head trauma (high-impact, fall, MVC, assault); brief loss of consciousness, lucid interval, headache, vomiting, focal neurological deficits, seizures; GCS score; anticoagulation/antiplatelet history

Section	Content Example
**Imaging Technique**	CT head without contrast (first-line); MRI if CT equivocal or for subacute/chronic evaluation (T1, T2, FLAIR, GRE/SWI, contrast if membranes suspected)
**Findings**	Location (right/left, temporal, parietal, frontal, occipital, posterior fossa); size (thickness, AP × CC); shape (biconvex/lens-shaped, limited by sutures); density (acute hyperdense, subacute iso/hypodense); associated mass effect (midline shift mm, sulcal effacement, ventricular compression, herniation signs); underlying skull fracture; associated lesions (contusion, SAH, SDH)
**Impression**	No EDH / Acute EDH / Subacute EDH. Include location, size, presence of fracture, and degree of mass effect.
**Recommendations**	Large EDH or with mass effect: emergent neurosurgical intervention. Small and asymptomatic: close observation and repeat imaging. Correct coagulopathy if present. ICU-level monitoring if unstable.

Table 2. Application in a synthetic normal case

Section	Content Example
**Clinical Information**	25-year-old male, minor head trauma during sports, GCS 15, no neuro deficit
**Imaging Technique**	CT brain without contrast
**Findings**	No extra-axial collection; normal ventricles and sulci; no skull fracture
**Impression**	No imaging evidence of epidural hematoma.
**Recommendations**	Symptomatic management; discharge with head trauma precautions.

Table 3. Application in a synthetic indeterminate case

Section	Content Example
**Clinical Information**	40-year-old female, head injury after fall, headache, nausea, mild confusion
**Imaging Technique**	CT brain without contrast
**Findings**	Right parietal convexity biconvex extra-axial collection measuring 7 mm in thickness, iso-to-hypodense compared to brain parenchyma (subacute stage), associated linear fracture of right parietal bone, minimal sulcal effacement, no midline shift
**Impression**	Small right subacute epidural hematoma with associated skull fracture, no significant mass effect.
**Recommendations**	Clinical observation; neurosurgical consultation; repeat CT in 24–48 hours.

Table 4. Application in a synthetic complicated case

Section	Content Example
**Clinical Information**	18-year-old male, motorcycle accident, brief loss of consciousness followed by lucid interval, now GCS 10 with left-sided weakness
**Imaging Technique**	CT brain without contrast
**Findings**	Large acute right temporoparietal epidural hematoma, biconvex hyperdense collection measuring 25 mm in maximum thickness and 8 cm length, causing 15 mm leftward midline shift, compression of right lateral ventricle, effacement of basal cisterns. Associated right temporal bone fracture crossing middle meningeal artery groove.
**Impression**	Large acute right temporoparietal epidural hematoma with severe mass effect and midline shift. Skull fracture present.
**Recommendations**	Emergent neurosurgical evacuation; ICU admission; monitor for herniation.

S38- Structured Reporting Template for Cerebral Venous Thrombosis

Table 1. Structured Reporting Template for Cerebral Venous Thrombosis

Section	Content Example
**Clinical Information**	Age, sex; risk factors (postpartum, OCP use, dehydration, thrombophilia, infection, malignancy, head trauma, neurosurgery); symptoms (headache, seizures, focal deficit, papilledema, altered consciousness)

Section	Content Example
**Imaging Technique**	CT brain without contrast; CT venography (CTV) with contrast; MRI brain with MR venography (2D TOF, contrast-enhanced); sequences: T1, T2, FLAIR, GRE/SWI (to detect thrombus and hemorrhage)
**Findings**	Venous sinus involved (superior sagittal, transverse, sigmoid, straight sinus, cortical veins, deep venous system); appearance of thrombus (hyperdense sinus on NCCT, filling defect on CTV/MRV); parenchymal changes (venous infarct, hemorrhagic transformation, edema); mass effect (midline shift, herniation signs); collateral circulation
**Impression**	No evidence of CVT / CVT in specified sinus (acute, subacute, chronic) ± venous infarction/hemorrhage
**Recommendations**	Initiate anticoagulation unless contraindicated (ICH does not always contraindicate); neurology/neurosurgical consultation; repeat imaging for progression; consider thrombophilia work-up

Table 2. Application in a synthetic normal case

Section	Content Example
**Clinical Information**	28-year-old female, postpartum day 5, headache
**Imaging Technique**	MRI brain with MRV
**Findings**	Normal flow-related signal in all dural venous sinuses and cortical veins; no parenchymal edema, infarction, or hemorrhage
**Impression**	No imaging evidence of cerebral venous thrombosis.
**Recommendations**	Clinical follow-up; exclude other causes of headache.

Table 3. Application in a synthetic indeterminate case

Section	Content Example
**Clinical Information**	45-year-old male, prolonged immobilization, headache and focal seizure
**Imaging Technique**	CT venography
**Findings**	Filling defect in left transverse sinus (non-opacified segment measuring 2 cm); no associated hemorrhage; mild vasogenic edema in adjacent left temporal lobe; no midline shift
**Impression**	Left transverse sinus thrombosis with mild adjacent venous edema.
**Recommendations**	Initiate anticoagulation; monitor clinically; repeat MRV in 5–7 days.

Table 4. Application in a synthetic complicated case

Section	Content Example
**Clinical Information**	32-year-old female, OCP use, sudden severe headache, seizure, altered consciousness
**Imaging Technique**	MRI brain with MRV
**Findings**	Extensive thrombosis of superior sagittal sinus and right transverse sinus with loss of flow void and absence of enhancement; large right frontal-parietal hemorrhagic venous infarct with surrounding edema; 8 mm midline shift to the left; effacement of right lateral ventricle and basal cisterns
**Impression**	Extensive superior sagittal and right transverse sinus thrombosis with large hemorrhagic venous infarct and significant mass effect.
**Recommendations**	Immediate anticoagulation unless contraindicated; ICU admission; neurology and neurosurgical consultation; consider decompressive surgery if mass effect progresses.

S39- Structured Reporting Template for Orbital Cellulitis

Table 1. Structured Reporting Template for Orbital Cellulitis

Section	Content Example
**Clinical Information**	Age, sex; recent sinus infection or trauma; immunocompromised status; symptoms: fever, periorbital pain, eyelid swelling, proptosis, decreased ocular motility, vision changes

Section	Content Example
**Imaging Technique**	CT orbit with IV contrast (preferred in acute setting); MRI orbit with contrast and fat-suppression (for soft tissue, intracranial extension); sequences: T1, T2, STIR, DWI, post-contrast
**Findings**	- **Preseptal vs Postseptal involvement** (key for differentiation)<br>- Orbital fat stranding / edema<br>- Extraocular muscle thickening / enhancement<br>- Subperiosteal abscess formation<br>- Sinus disease (ethmoid/sphenoid involvement)<br>- Proptosis degree<br>- Optic nerve involvement (edema, enhancement)<br>- Intracranial extension (cavernous sinus thrombosis, meningitis, epidural/subdural empyema)
**Impression**	No orbital cellulitis / Orbital cellulitis with extent (preseptal, postseptal, abscess, intracranial extension)
**Recommendations**	IV antibiotics; ENT/ophthalmology consultation; urgent surgical drainage if abscess or vision-threatening complications; repeat imaging if clinical worsening

Table 2. Application in a synthetic normal case

Section	Content Example
**Clinical Information**	12-year-old boy, sinusitis, eyelid swelling
**Imaging Technique**	CT orbit with contrast
**Findings**	Preseptal soft tissue swelling without postseptal extension; orbital fat preserved; extraocular muscles normal; no abscess; paranasal sinuses with mucosal thickening
**Impression**	Preseptal cellulitis, no evidence of orbital cellulitis.
**Recommendations**	Medical management with IV antibiotics; ENT follow-up.

Table 3. Application in a synthetic indeterminate case

Section	Content Example
**Clinical Information**	34-year-old female, fever, proptosis, decreased eye movement
**Imaging Technique**	MRI orbit with contrast
**Findings**	Postseptal orbital fat stranding with mild thickening and enhancement of medial rectus muscle; ethmoid sinusitis; no discrete abscess or cavernous sinus involvement
**Impression**	Postseptal orbital cellulitis, no abscess formation or intracranial extension.
**Recommendations**	IV antibiotics, close ophthalmology/ENT follow-up; repeat imaging if no clinical improvement.

Table 4. Application in a synthetic complicated case

Section	Content Example
**Clinical Information**	8-year-old boy, high fever, severe proptosis, vision loss, lethargy
**Imaging Technique**	CT orbit with IV contrast
**Findings**	Large subperiosteal abscess along medial orbit with rim enhancement; severe proptosis; compression of optic nerve; diffuse ethmoid and sphenoid sinusitis; extension to cavernous sinus with filling defect suggesting thrombosis
**Impression**	Orbital cellulitis with large medial subperiosteal abscess and cavernous sinus thrombosis.
**Recommendations**	Urgent surgical drainage; IV broad-spectrum antibiotics; ICU monitoring; neurosurgical and ENT consultation.

S40- Structured Reporting Template for Foreign Body Aspiration

Table 1. Structured Reporting Template for Foreign Body Aspiration

Section	Content Example
**Clinical Information**	Age, sex; choking episode; cough, stridor, wheezing, recurrent pneumonia; unilateral decreased breath sounds; history of neurologic impairment or aspiration risk
**Imaging Technique**	Chest radiograph (inspiration/expiration views); CT chest (low-dose, non-contrast; preferred if X-ray inconclusive); Virtual bronchoscopy reconstruction (if available); Fluoroscopy (dynamic airway evaluation)

Section	Content Example
Findings	- **Foreign body visibility** (radiopaque or radiolucent)<br>- Location (trachea, mainstem bronchus, lobar bronchus, distal airway)<br>- Degree of airway obstruction (partial/complete)<br>- Secondary findings: air-trapping, atelectasis, consolidation, hyperinflation, mediastinal shift<br>- Complications: post-obstructive pneumonia, bronchiectasis
Impression	No evidence of foreign body / Foreign body aspiration with location and secondary changes
Recommendations	Urgent bronchoscopy for removal if confirmed or highly suspected; supportive care (antibiotics if pneumonia, oxygen if needed); follow-up imaging post-removal

Table 2. Application in a synthetic normal case

Section	Content Example
Clinical Information	5-year-old boy, sudden cough after eating peanuts, mild wheezing
Imaging Technique	Chest X-ray (PA, lateral)
Findings	No visible foreign body; both lungs clear; no focal atelectasis or air-trapping
Impression	No radiographic evidence of foreign body aspiration. Clinical suspicion remains.
Recommendations	Consider bronchoscopy if clinical suspicion persists.

Table 3. Application in a synthetic indeterminate case

Section	Content Example
Clinical Information	2-year-old girl, choking episode, persistent cough
Imaging Technique	Chest X-ray (inspiration/expiration)
Findings	No radiopaque foreign body; right lung hyperinflation with mediastinal shift to left on expiratory film; no consolidation
Impression	Findings suspicious for non-radiopaque foreign body in right main bronchus causing partial obstruction.
Recommendations	Diagnostic and therapeutic bronchoscopy advised.

Table 4. Application in a synthetic complicated case

Section	Content Example
Clinical Information	45-year-old male with seizure disorder, fever, cough, dyspnea
Imaging Technique	CT chest (non-contrast)
Findings	Hyperdense foreign body lodged in left main bronchus; complete obstruction with collapse of left lower lobe; post-obstructive consolidation; small left pleural effusion
Impression	Left main bronchus foreign body with lobar collapse and post-obstructive pneumonia.
Recommendations	Urgent bronchoscopy for removal; IV antibiotics; close respiratory monitoring.
